# STMGraph: spatial-context-aware of transcriptomes via a dual-remasked dynamic graph attention model

**DOI:** 10.1093/bib/bbae685

**Published:** 2025-01-06

**Authors:** Lixian Lin, Haoyu Wang, Yuxiao Chen, Yuanyuan Wang, Yujie Xu, Zhenglin Chen, Yuemin Yang, Kunpeng Liu, Xiaokai Ma

**Affiliations:** Center for Genomics and Biotechnology, Fujian Provincial Key Laboratory of Haixia Applied Plant Systems Biology, Haixia Institute of Science and Technology, Fujian Agriculture and Forestry University, No. 15 Shangxiadian Road, Cangshan District, Fuzhou 350002, China; College of Life Science, Fujian Agriculture and Forestry University, No. 15 Shangxiadian Road, Cangshan District, Fuzhou 350002, China; Center for Genomics and Biotechnology, Fujian Provincial Key Laboratory of Haixia Applied Plant Systems Biology, Haixia Institute of Science and Technology, Fujian Agriculture and Forestry University, No. 15 Shangxiadian Road, Cangshan District, Fuzhou 350002, China; Center for Genomics and Biotechnology, Fujian Provincial Key Laboratory of Haixia Applied Plant Systems Biology, Haixia Institute of Science and Technology, Fujian Agriculture and Forestry University, No. 15 Shangxiadian Road, Cangshan District, Fuzhou 350002, China; Center for Genomics and Biotechnology, Fujian Provincial Key Laboratory of Haixia Applied Plant Systems Biology, Haixia Institute of Science and Technology, Fujian Agriculture and Forestry University, No. 15 Shangxiadian Road, Cangshan District, Fuzhou 350002, China; Center for Genomics and Biotechnology, Fujian Provincial Key Laboratory of Haixia Applied Plant Systems Biology, Haixia Institute of Science and Technology, Fujian Agriculture and Forestry University, No. 15 Shangxiadian Road, Cangshan District, Fuzhou 350002, China; Center for Genomics and Biotechnology, Fujian Provincial Key Laboratory of Haixia Applied Plant Systems Biology, Haixia Institute of Science and Technology, Fujian Agriculture and Forestry University, No. 15 Shangxiadian Road, Cangshan District, Fuzhou 350002, China; Center for Genomics and Biotechnology, Fujian Provincial Key Laboratory of Haixia Applied Plant Systems Biology, Haixia Institute of Science and Technology, Fujian Agriculture and Forestry University, No. 15 Shangxiadian Road, Cangshan District, Fuzhou 350002, China; College of Life Science, Fujian Agriculture and Forestry University, No. 15 Shangxiadian Road, Cangshan District, Fuzhou 350002, China; Center for Genomics and Biotechnology, Fujian Provincial Key Laboratory of Haixia Applied Plant Systems Biology, Haixia Institute of Science and Technology, Fujian Agriculture and Forestry University, No. 15 Shangxiadian Road, Cangshan District, Fuzhou 350002, China; Center for Genomics and Biotechnology, Fujian Provincial Key Laboratory of Haixia Applied Plant Systems Biology, Haixia Institute of Science and Technology, Fujian Agriculture and Forestry University, No. 15 Shangxiadian Road, Cangshan District, Fuzhou 350002, China; Key Laboratory of Orchid Conservation and Utilization of National Forestry and Grassland Administration, Fujian Agriculture and Forestry University, No. 15 Shangxiadian Road, Cangshan District, Fuzhou 350002, China

**Keywords:** spatial transcriptomics, dynamic graph attention model, dual-remask, microenvironmental heterogeneity detecting, spatial domain clustering, batch-effects correction

## Abstract

Spatial transcriptomics (ST) technologies enable dissecting the tissue architecture in spatial context. To perceive the global contextual information of gene expression patterns in tissue, the spatial dependence of cells must be fully considered by integrating both local and non-local features by means of spatial-context-aware. However, the current ST integration algorithm ignores for ST dropouts, which impedes the spatial-aware of ST features, resulting in challenges in the accuracy and robustness of microenvironmental heterogeneity detecting, spatial domain clustering, and batch-effects correction. Here, we developed an STMGraph, a universal dual-view dynamic deep learning framework that combines dual-remask (MASK-REMASK) with dynamic graph attention model (DGAT) to exploit ST data outperforming pre-existing tools. The dual-remask mechanism masks the embeddings before encoding and decoding, establishing dual-decoding-view to share features mutually. DGAT leverages self-supervision to update graph linkage relationships from two distinct perspectives, thereby generating a comprehensive representation for each node. Systematic benchmarking against 10 state-of-the-art tools revealed that the STMGraph has the optimal performance with high accuracy and robustness on spatial domain clustering for the datasets of diverse ST platforms from multi- to sub-cellular resolutions. Furthermore, STMGraph aggregates ST information cross regions by dual-remask to realize the batch-effects correction implicitly, allowing for spatial domain clustering of ST multi-slices. STMGraph is platform independent and superior in spatial-context-aware to achieve microenvironmental heterogeneity detection, spatial domain clustering, batch-effects correction, and new biological discovery, and is therefore a desirable novel tool for diverse ST studies

## Introduction

In multicellular organisms, cells typically exist in the form of physically aggregated groups of similar cells within tissues. In order to better dissect the emergent properties and pathology of tissues by linking cellular gene expression with their spatial distribution [1, 2], the spatial transcriptomics (ST) technology was developed [3]. Currently, commonly used ST sequencing techniques include 10× Genomics Visium [4], Slide-seq [5], Stereo-seq [6, 7], STARmap [8], DBiT-seq [9], and Seq-Scope [10] ranging from multicellular (10× Genomics Visium) to subcellular (STARmap, Stereo-seq, Slide-seq, Seq-Scope, and DBiT-seq) resolutions. Despite the ongoing advancements in ST sequencing technology, ST-context-aware tasks with perceiving the contextual information of gene expression patterns are frequently impeded by the presence of impure data [11], which hinders the identification of spatial domains; this will also influence the efficacy of subsequent downstream comparative analysis, such as gene denoising [12], trajectory inference [13], and spatially differentially expressed gene (SDEG) [14] detections.

The methods for spatial domain identification mainly employ the gene expression data. The commonly used clustering methods include K-means [15], Louvain [16, 17], and Leiden [17, 18], but they lack consideration for spatial location. Recently, many ST clustering methods have been developed, which integrate spatial information and gene expression profiles. BayesSpace [19] uses spatial neighborhood information to enhance the resolution of ST data through statistical methods and perform cluster analysis. SpaGCN [20] and DeepST [21], based on GCN [22], both are able to integrate omics data and histological images for clustering, while DeepST quantifies images by using pretrained neural network rather than SpaGCN of using weighted sum of the RGB values. Nevertheless, a simple addition of images information to the omics data also can be deleterious to the seamless integration of spatial information [23]. STAGATE [24] utilizes GAT [25] to perceive the similarities and differences between its own points and adjacent points, achieving spatial domain partitioning. Although it no longer requires calculating graph weights, the lack of accurate weight calculation is considered static graph attention [25]. In addition, currently popular software for ST domain clustering primarily combine omics and spatial location information through graph convolutional neural networks [22, 25, 26], which can be neatly divided into two distinct categories: generative graph self-supervised learning (Graph SSL) and contrastive graph self-supervised learning [27]. Notably, DeepST [21], STAGATE [24], and Spatial-MGCN [28, 29] are classified under the generative Graph SSL. Conversely, GraphST [30], conST [31], and SpaceFlow [32] are recognized as part of the contrastive Graph SSL. However, there are often errors in the subgraph partitioning of contrastive Graph SSL [33, 34]. The current ST clustering methods face the challenge of introducing noise into the embeddings from weak graph attention network and ST unmeasured spots [11, 12, 23, 35], limiting the spatial-context-awareness of these models.

The software mentioned above concerns with the differences between organizations, but often neglects the nuanced variations within individual microenvironment. Although STAGATE employs GAT to capture the heterogeneous similarity among spots in microenvironments, this attention mechanism is inherently weak and contingent upon pre-clustering (Leiden or Louvain). The accuracy of this pre-clustering step is frequently uncertain, making it especially critical to enhance the spatial awareness of attention mechanisms to improve the fidelity of spot differentiation. Owing to the limitation of ST sequencing chips for the capture tissue area, samples are often segmented into multiple slices [30]. To fully detect the entire region of interest, adjacent slices must be jointly analyzed to accurately identify various tissue organs vertically and horizontally. In addition, batch-effects correction methods developed for scRNA-seq data, such as Harmony [36], are not suitable for spatial domain identification because they only consider gene expression and do not utilize associated spatial information. Although current ST clustering algorithm such as GraphST has batch-correction ability, the accuracy of multi-slice joint clustering is still poor due to the limitation of contrastive Graph SSL. Currently, clustering algorithms based on generative graph SSL lack the capability to implicitly correct vertical slice effects.

To address the aforementioned limitations and challenges, we propose a dual-remask (MASK-REMASK) dynamic graph attention [37] model (DGAT) based on Generative Graph SSL, called STMGraph. Generated latent embeddings from STMGraph are used for microenvironmental heterogeneity detection, spatial domain clustering, and batch-effects correction. To ensure that the most relevant information is prioritized, we use DGAT to adaptively weigh the contributions of different nodes. The priority weight was also documented to assess the diversity of the microenvironment within each community simultaneously. The dual-view remasked algorithm masks ST embeddings in a high proportion and captures features from different angles by self-supervision to reduce the interference of ST dropouts. In addition, DGAT infers masked embeddings by aggregating information from different batch regions implicitly, effectively alleviating batch-effects in joint analysis of multi-slices. Furthermore, STMGraph has achieved the optimal clustering performance across datasets of different ST platform such as 10× Genomics Visium, Slide-seq, Stereo-seq, and STARmap with diverse resolutions. In summary, STMGraph is a novel model that exhibits versatility and multi-platform compatibility when contrasted with the current state-of-the-art tools available in the market.

## Methods

### STMGraph overview

**Figure 1:**
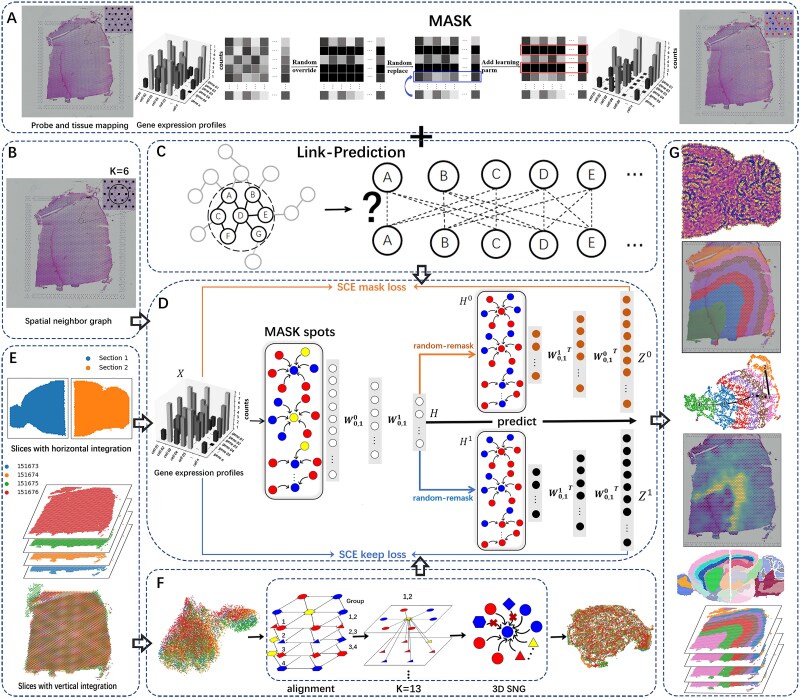
Overview of STMGraph. (A) The MASK process involves randomly selecting a certain number of spots from the valid spots data for labeling. The labeled spots are then subjected to random replacement or incorporation of trainable parameters. (B) After mapping the gene probe to the sliced tissue, each spot will construct a spatial neighbor graph (SNG). (C) Using dynamic graph attention for graph link prediction. (D) Before each training session, we utilize the MASK algorithm to modify the original transcriptomic information. The MASK algorithm divides points into three groups. The algorithm randomly discard the transcriptomic information of 50% of the points, five percent of the points are randomly selected from the discarded points, and then 5% of the points are randomly chosen from the original spots to replace them. The remaining 50% of the points undergo no processing, i.e. they represent the original gene probe information obtained by measurement. After the application of the mask, DGAT is utilized to capture ST data and establish relationships between its points. The lowest-dimensional embeddings are then random-remask twice, with one view reconstructing discarded points and the other view reconstructing unchanged nodes. (E) Integrating and aligning horizontal and vertical slices. (F) 3DSNG construction of vertical alignment and batch correction. The numbers of the four layers of slices are 1 to 4 from the top down, and the node shapes are circular, triangle, diamond, and hexagon from top to bottom. The nearest neighbor of the topmost circular node is *k* = 6 + 7, where 6 indicates the number of nearest neighbors on the same slice and 7 indicates the number of neighbor nodes that are not on the same slice as the circular node but are on the nearest slice (where the neighbor is triangle nodes), excluding the penultimate diamond and the bottommost hexagonal nodes. (G) STMGraph obtains the results of spot heterogeneity relationships, spatial domain clustering, PAGA (partition-based graph abstraction), gene denoising, as well as integrating horizontal and vertical slices for spatial domain identification.

**Figure 2:**
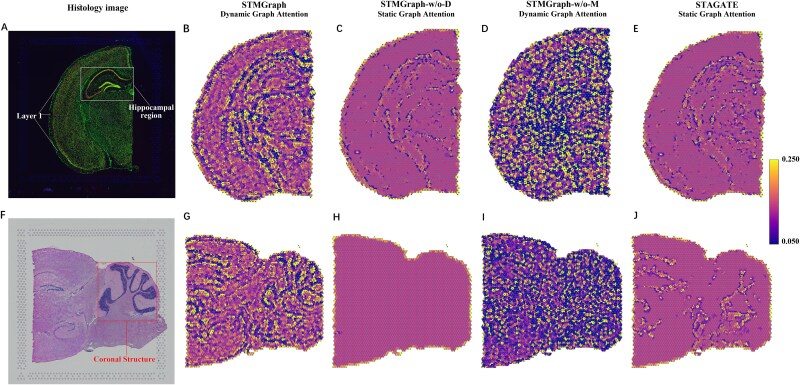
STMGraph demonstrates superior ability in detecting the spatial heterogeneous similarities within tissue microenvironment of 10× Visium dataset. (A) Immunofluorescent image of the adult coronal mouse brain section stained with DAPI and anti-NeuN. (F) Histological image of the adult mouse posterior brain tissue section. Visualizations of the attention layers detected by STMGraph (B, G), STMGraph-w/o-D (C, H), STMGraph-w/o-M (D, I), and STAGATE (E, J). The nodes of the attention layer are arranged according to the spatial position of spots. The edges of the attention layer are colored by corresponding weights.

**Figure 3:**
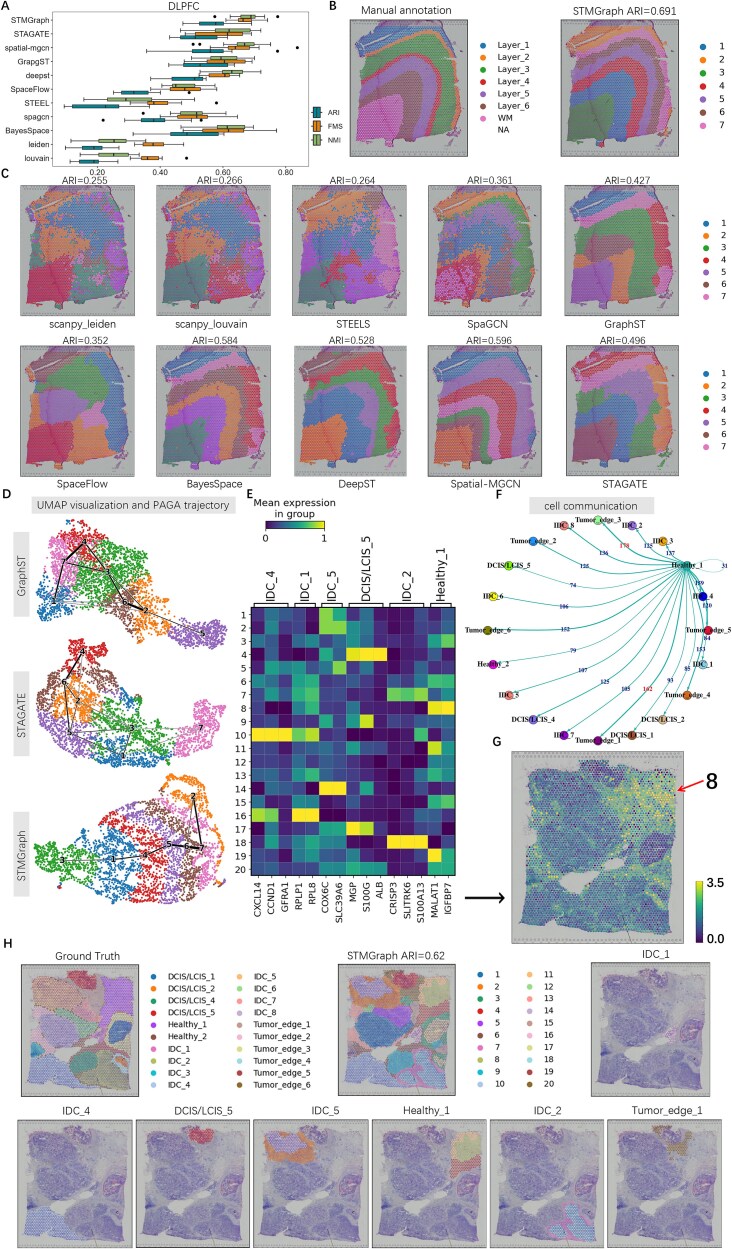
Benchmark test of STMGraph against with 10 state-of-the-arts tools based on 10× ST platform. (A) Comparison of clustering accuracy in all 12 sections of the DLPFC dataset in terms of ARI, FMS, and NMI scores for 11 methods. In the boxplot, the center line, box limits, and whiskers denote the median, upper and lower quartiles, and 1.5× interquartile range, respectively. (B) Ground-truth segmentation of cortical layers and white matter (WM) in the DLPFC section 151674. (C) Cluster assignments generated by STMGraph, SCANPY, STEEL, SpaGCN, GraphST, SpaceFlow, BayesSpace, DeepST, spatial-MGCN, and STAGATE in the DLPFC section 151674. (D) UMAP visualizations and PAGA graphs generated by STMGraph, GraphST, and STAGATE embeddings, respectively, in the DLPFC section 151674. (E) The expression heatmaps of the structural domains on the top three DEGs from clusters IDC_1, IDC_2, IDC_4, IDC_5, DCIS/LCIS_5, and healthy_1. (F) The communication between healthy cells and tumor cells in human breast cancer data is represented by the size of the link, indicating the strength of their interaction. (G) The spatial expression pattern of *IGFBP7*. (H) Human breast cancer samples stained with H&E were annotated and STMGraph clustering results were performed. Visualization of each spatial domain identified by STMGraph.

STMGraph first uses dynamic graph attention model (DGAT) to aggregate neighboring ST context information and reduces the interference of noisy neighbors on the ST-context-aware, enhancing the signal-to-noise ratio of latent embedding. Secondly, dual-remask reduces overfitting of input ST features and predict potential representations with more informative ST targets during decoding. Finally, it uses SCE (scale cosine error) as the loss function to correct latent embedding through self-supervision. It adopts a high proportion of random MASK strategy to optimize the feature propagation process in the graph structure (Fig. 1A). It contributes to the revelation of the characteristics and functions of different regions within tissues, providing clues for understanding cellular interactions, particularly signaling between neighboring cells. The framework comprises three modules: (1) heterogeneous similarity detection of microenvironment (Fig. 1C and G), (2) spatially informed clustering (Fig. 1D and G), and (3) batch-effects correction (Fig. 1E and F).

### Data preprocessing

In all datasets (Supplementary Table S1), we initially excluded spots located outside the central tissue region. Next, we performed a log transformation and normalized the raw gene expression. We pre-filtered exogenous RNAs and mitochondrial genes, filtered out genes with expression in less than three cells, and selected the top 3000 highly variable genes. For denoising gene expression profiles, selecting the top 3000 variable genes is unnecessary. The above operation is based on the Scanpy package [18].

### Construction of SNG

We constructed a binary adjacency matrix $(A)$ by considering each point as the center and including the points within a certain radius to form a SNG, then ${A}_{ij}=1$ if and only if the Euclidean distance between spot *i* and spot *j* is less than radius. In the ST sequencing method at multicellular resolution, variations in data resolution may result in differences in interpoint distances, but the spatial arrangement of spots remains consistent. For instance, on a 10× platform, each point is surrounded by 6 nearest neighbors (Fig. 1B). In this case, we use KNN (K-nearest neighbors) [38] to automatically capture the minimum radius covering K neighbors. For single-cell level resolution ST data, the SNG is created by similar manner as Spatial Neighbor Network (SNN) to construct the adjacency matrix [24], utilizing a predetermined radius. The SNG translated spatial information into an undirected network of neighboring points. In addition, to analyze the ST data of horizontal and vertical structure, we constructed a 2DSNG and 3DSNG, respectively. The 2DSNG can compute the neighborhood of each point in two horizontally aligned slices. However, when it comes to three or more slices, it consolidates points from nonadjacent slices into the neighborhood. To ensure that each point builds a neighborhood that includes only the nearest point in the horizontal and vertical directions, we build 3DSNG, which remains based on the nearest neighbor algorithm. Therefore, in addition to calculating the nearest neighbor of its own slice, it also includes the adjacent slice nodes closest to its own slice, and the remaining slice nodes are not considered in the 3DSNG range. After PASTE [39] aligns and merges the slices, 3DSNG first divides the four slices into three groups (1, 2), (2, 3), and (3, 4) and scans them from top to bottom. The method of calculating nearest neighbor nodes for each group can be converted to 2DSNG. The nodes closest to itself in the same slice are at most 6, and the nearest neighbors to itself in adjacent slices are at most 7. Slices #1 and #4 have only one nearest-neighbor slice covering at most 6 + 7 nearest points, and the nodes in slices #2 and #3 have two nearest-neighbor slices covering at most 6 + 7 + 7 nearest points. Slice #2 in group (1, 2) has already computed its nearest neighbor to slice #1, and slice #2 in group (2, 3) computes its nearest neighbor to slice #3 so that slice #2 does not miss its nearest neighbor, while slice #3 computes its nearest neighbor in the same way (Fig. 1F).

### STMGraph structure

The structure of the STMGraph was divided into three parts: (1) MASK-REMASK definition, (2) DGAT construction, and (3) Dual-view remask operation rules. First, we divide the features into two subsets: “mask” and “keep.” ***X*** is the original matrix, where $m\in \mathrm{mask},k\in \mathrm{keep}$. ***H*** originates from the latent embedding in the encoder, which is obtained through two rounds of REMASK to generate spatial embedding ${\boldsymbol{H}}^0$ and ${\boldsymbol{H}}^1$, respectively. ${\boldsymbol{H}}^0$ is decoded by the first decoder to obtain ${\boldsymbol{Z}}^0$. Similarly, ${\boldsymbol{H}}^1$ is decoded by the second decoder to obtain ${\boldsymbol{Z}}^1$. The ***X*** matrix is reconstructed by dividing it into two different parts (mask and keep). Node ${\boldsymbol{Z}}_m^0$ is the reconstructed nodes of ${\boldsymbol{X}}_m$. Node ${\boldsymbol{Z}}_k^1$ is the node reconstructed by ${\boldsymbol{X}}_k$ (Fig. 1D). Lastly, the SCE (scaled cosine error) is chosen to calculate the loss for the keep subset and mask subset.

(1) MASK-REMASK

Based on the pre-constructed SNG, before each training session, we utilize the MASK algorithm to modify the original transcriptomic information. $\tilde{\boldsymbol{X}}$ = $\mathrm{MASK}\left(\boldsymbol{X}\right)$: Given a graph *G* = (*V*, *A*, *X*), *V* is the set of nodes, $N=\mid \boldsymbol{V}\mid$ is the number of nodes, $\boldsymbol{A}\in{\left\{0,1\right\}}^{\mathrm{N}\times \mathrm{N}}$ is SNG, and $\boldsymbol{X}\in{\mathbb{R}}^{N\times{d}_{in}}$ is the gene expression profiles matrix. Randomly masking 50% nodes in *V*$,$ the masked nodes are divided into two parts. A small portion of nodes (5%) is randomly selected from the masked nodes and replaced with nodes from the global nodes. The replacement rate of microenvironment heterogeneity detecting is 0%. The other portion of nodes is augmented with trainable parameters [40, 41]. Formally, we sample a subset of nodes $\tilde{\boldsymbol{V}}\subset \boldsymbol{V}$ and mask each of their features with a mask token [MASK], i.e. a learnable vector ${\boldsymbol{x}}_{\left[M\ \right]}\in{\mathbb{R}}^{d_{in}}$ (Fig. 1A). Thus, the node feature ${\tilde{\boldsymbol{x}}}_i$ for ${\tilde{\boldsymbol{x}}}_i\in \boldsymbol{V}$ in the masked feature matrix $\tilde{\boldsymbol{x}}$ can be defined as


$$ {\tilde{\boldsymbol{x}}}_{\boldsymbol{i}}=\left\{\begin{array}{c}{\boldsymbol{x}}_{\left[M\ \right]}\kern2.25em {v}_i\in \tilde{\boldsymbol{V}}\ \\{}{\boldsymbol{x}}_i\kern3.25em {v}_i\notin \tilde{\boldsymbol{V}}\end{array}\right. $$

$\boldsymbol{X}=\mathrm{KEEP}\left(\boldsymbol{X}\right)$ : Unmasked nodes do not undertake any modifications.

$\tilde{\boldsymbol{H}}=\mathrm{REMASK}\left(\boldsymbol{H}\right)$ : The decoder needs to randomly remasked embedding $\boldsymbol{H}\boldsymbol{\in }{\mathbb{R}}^{N\times d}$ before decoding it. Randomly mask 50% of nodes $\overline{\boldsymbol{V}}$, following the same MASK rule except for random replacement. Formally, we resample a subset of nodes $\overline{\boldsymbol{V}}\subset \boldsymbol{V}$ following a uniform distribution. $\overline{\boldsymbol{V}}$ is different from the input masked nodes $\tilde{\boldsymbol{V}}$ and nodes are equally selected for remasking regardless of whether they are masked before [42]. Then, corrupted representation matrix $\tilde{\boldsymbol{H}}$ is built from ${\boldsymbol{H}}$ by replacing the ${\boldsymbol{h}}_{\boldsymbol{i}}$ of node ${v}_i\in \overline{\boldsymbol{V}}$ with another shared mask token [DMASK], i.e. a learnable vector ${\boldsymbol{h}}_{\left[\mathbf{M}\right]}\in{\mathbb{R}}^d$:


$$ {\tilde{\boldsymbol{h}}}_{\boldsymbol{i}}=\left\{\begin{array}{c}{\boldsymbol{h}}_{\left[M\ \right]}\kern2.25em {v}_i\in \overline{\boldsymbol{V}}\ \\{}{\boldsymbol{h}}_i\kern3.25em {v}_i\notin \overline{\boldsymbol{V}}\end{array}\right. $$

(2) Dynamic graph attention model (DGAT)

Encoder $\boldsymbol{H}={\mathrm{f}}_{\mathrm{E}}\left(\boldsymbol{A},\boldsymbol{X}\right),\kern0.5em \boldsymbol{H}\boldsymbol{\in }{\mathbb{R}}^{N\times d}$, the encoder takes normalized gene expressions as input and generates spot embeddings by collectively aggregating information from its neighbors. Let ${\boldsymbol{x}}_{\boldsymbol{i}}$ be the normalized expressions of spot *i* and *L* be the number of layers of the encoder. By treating expression profiles as initial spot embeddings $\left(\mathrm{i}.\mathrm{e}.\kern0.75em {\boldsymbol{h}}_i^{(0)}={\boldsymbol{x}}_{\boldsymbol{i}},\kern0.5em \forall i\in \left\{1,2,3, \ldots, N\right\}\right),\mathrm{the}\ k-\mathrm{th}\ \left(k\in \left\{1,2,\ldots,L-1,L\right\}\right)$ encoder layer generates the embedding of spot *i* in layer *k* as follows:


$$ {\boldsymbol{h}}_i^{(k)}=\mathrm{ELU}\left(\sum_{j\in{N}_i}{\boldsymbol{att}}_{\boldsymbol{ij}}^{\left(\boldsymbol{k}\right)}\left({\boldsymbol{W}}_0^{(k)}{\boldsymbol{h}}_j^{\left(k-1\right)}\right)\right) $$

${\boldsymbol{W}}_{\mathbf{0}},{\boldsymbol{W}}_{\mathbf{1}}$ are two trainable matrices. The feature vector of each node is linearly transformed through ${\boldsymbol{W}}_{\mathbf{0}}$, which helps the model learn a higher-level representation of the node feature. ELU is a nonlinear activation function. ${N}_i$ represents the neighborhood set of point *i* in SNG, including point *i* itself. ${\boldsymbol{att}}_{\boldsymbol{ij}}^{\left(\boldsymbol{k}\right)}$ denotes the edge weight between point *i* and point *j* in the output of the *k*-th dynamic graph attention layer. The *L*-th encoder is calculated without the attention mechanism as follows:


$$ {\boldsymbol{h}}_i^{(L)}=\mathrm{ELU}\left(\sum_{\mathrm{j}\in{N}_i}\left({\boldsymbol{W}}_0^{(L)}{\boldsymbol{h}}_j^{\left(L-1\right)}\right)\right) $$

Decoder $\boldsymbol{Z}={\mathrm{f}}_{\mathrm{D}}\left(\boldsymbol{A},\boldsymbol{H}\right),$ where $\boldsymbol{Z}$ denotes the reconstructed gene expression matrix. The training weight of the decoder is the transpose of the training weight of the encoder. By processing the output of the encoder as input to the decoder $\left(\mathrm{i}.\mathrm{e}.{\hat{\boldsymbol{h}}}_i^{(L)}={\boldsymbol{h}}_i^{(L)}\right),\mathrm{the}\ k-\mathrm{th}\ \left(k\in \left\{2,\ldots, L-1,L\right\}\right)$ decoder layer reconstructs the layer *k* − 1 as follows:


$$ {\hat{\boldsymbol{h}}}_i^{\left(k-1\right)}=\mathrm{ELU}\left(\sum_{\mathrm{j}\in{N}_i}{\hat{\boldsymbol{att}}}_{ij}^{\left(k-1\right)}\left({\hat{\boldsymbol{W}}}_0^{(k)}{\hat{\boldsymbol{h}}}_j^{(k)}\right)\right) $$

Like the encoder, there is no need to add attention to the last layer:


$$ {\hat{\boldsymbol{h}}}_i^{(0)}=\mathrm{ELU}\left(\sum_{\mathrm{j}\in{N}_i}\left({\hat{\boldsymbol{W}}}_0^{(1)}{\hat{\boldsymbol{h}}}_j^{(1)}\right)\right) $$

The output of decoder is considered as the reconstructed normalized expressions. To avoid overfitting, STMGraph sets ${\hat{\boldsymbol{W}}}_0^{(k)^T}={\boldsymbol{W}}_0^{(k)}$, ${\hat{\boldsymbol{W}}}_1^{(k)^T}={\boldsymbol{W}}_1^{(k)}$, and ${\hat{\boldsymbol{att}}}^k={\boldsymbol{att}}^k$, respectively (Fig. 1D). Not only that, ${\boldsymbol{W}}_{\mathbf{0}},{\boldsymbol{W}}_{\mathbf{1}}$ capture interrelationships between nodes during training.

To adaptively learn the similarity spots between neighborhoods, we adopt a dynamic graph attention variant different from the static attention mechanism (GATv1) [25], which first uses activation functions and then uses attention operations (Fig. 1C). The attention variant is a single-layer feedforward neural network with shared parameter nodes and parameterized by weight vectors. In the *k*-th layer encoder, the edge weights from node *i* to its adjacent node *j* are calculated as follows:


$$ {\boldsymbol{e}}_{\boldsymbol{ij}}^{\left(\boldsymbol{k}\right)}={{\boldsymbol{v}}^{(k)}}^T\left[\mathrm{Sigmoid}\left({\boldsymbol{W}}_0{\boldsymbol{h}}_i^{\left(k-1\right)}+{\boldsymbol{W}}_1{\boldsymbol{h}}_j^{\left(k-1\right)}\right)\right] $$

${\boldsymbol{v}}^{\left(\boldsymbol{k}\right)}$ is a trainable parameter, and Sigmoid represents the sigmoid activation function. Attention can be calculated through the softmax function.


$$ {\boldsymbol{att}}_{ij}^{(k)}=\frac{\exp \left({\boldsymbol{e}}_{\boldsymbol{ij}}^{\left(\boldsymbol{k}\right)}\right)}{\sum_{\mathrm{k}\in{N}_i}\exp \left({\boldsymbol{e}}_{\boldsymbol{ij}}^{\left(\boldsymbol{k}\right)}\right)} $$

(3) Dual-view remask

The $L-\mathrm{th}$ hidden layer generated by the DGAT’s encoder, denoted as $\tilde{\boldsymbol{X}}=\mathrm{MASK}\left(\boldsymbol{X}\right),\boldsymbol{H}={\mathrm{f}}_{\mathrm{E}}\left(\boldsymbol{A},\tilde{\boldsymbol{X}}\right)$, will be decoded through two different perspectives. Both views will be randomly remasked to obtain two new embeddings, namely, ${\tilde{\boldsymbol{H}}}^{\mathbf{0}}=\mathrm{REMASK}\left(\boldsymbol{H}\right)$ and ${\tilde{\boldsymbol{H}}}^{\mathbf{1}}=\mathrm{REMASK}\left(\boldsymbol{H}\right)$. Finally, the two embeddings will be separately inputted into two decoders, resulting in two reconstructed feature matrices as outputs, denoted as ${\boldsymbol{Z}}^{\mathbf{0}}={\mathrm{f}}_{\mathrm{D}}\left(\boldsymbol{A},{\tilde{\boldsymbol{H}}}^{\mathbf{0}}\right)$ and ${\boldsymbol{Z}}^{\mathbf{1}}={\mathrm{f}}_{\mathrm{D}}\left(\boldsymbol{A},{\tilde{\boldsymbol{H}}}^{\mathbf{1}}\right)$ (Fig. 1D). Each round of training masked and remasked nodes are randomly assigned.

(4) Loss function

The objective of STMGraph is to minimize the reconstruction loss of normalized expressions by utilizing SCE, where the $\gamma$ hyperparameter is used to adjust the rate at which the loss descents [42]. For ST data, dropout events are common in nodes. To make the embedding learn richer representations, we use one view focusing on detail recovery to reconstruct the unmasked nodes and another view focusing on noise correction to predict the masked nodes, allowing masked nodes and kept nodes to mutually guide. Predicting masked nodes loss function as follows:


$$ {L}_{sce0}=1/\left|\tilde{\boldsymbol{V}}\right|\sum_{{\boldsymbol{v}}_i\in \tilde{\boldsymbol{V}}}{\left(1-\frac{{{\boldsymbol{x}}_{\boldsymbol{i}}}^{\boldsymbol{T}}{\boldsymbol{z}}_{\boldsymbol{i}}^{\mathbf{0}}}{\left\Vert{\boldsymbol{x}}_{\boldsymbol{i}}\right\Vert \cdotp \left\Vert{\boldsymbol{z}}_{\boldsymbol{i}}^{\mathbf{0}}\right\Vert}\right)}^{\gamma } $$

Reconstructing kept nodes loss function as follows:


$$ {L}_{sce1}=1/\left(N-\left|\tilde{\boldsymbol{V}}\right|\right)\sum_{v_i\notin \tilde{V}}{\left(1-\frac{{{\boldsymbol{x}}_{\boldsymbol{i}}}^{\boldsymbol{T}}{\boldsymbol{z}}_{\boldsymbol{i}}^{\mathbf{1}}}{\left\Vert{\boldsymbol{x}}_{\boldsymbol{i}}\right\Vert \cdotp \left\Vert{\boldsymbol{z}}_{\boldsymbol{i}}^{\mathbf{1}}\right\Vert}\right)}^{\gamma } $$

${L}_2$ regularization is added to improve model stability:


$$ {L}_{\mathrm{weight}\ \mathrm{decay}\ \mathrm{loss}}=\sum_{i=0}^1\kern0.1em WD\cdotp{\left\Vert{W}_i\right\Vert}_2^2 $$

Overall loss function:


$$ Loss={L}_{sce1}+{L}_{sce0}+{L}_{\mathrm{weight}\ \mathrm{decay}\ \mathrm{loss}} $$

To train the STMGraph, a two-layer encoder of DGAT with dimensions (512, 30) is used, which allows each spot to consider second-order neighbor information. The parameter γ is varied within the range of (1, 3) [42]. The training process employs the Adam optimizer with a learning rate of 0.001 and a WD (weight decay) set to 0.001 (see detailed hyperparameters in supplementary Table S2).

### Link prediction

The ${\boldsymbol{att}}_{\boldsymbol{ij}}^{\left(\boldsymbol{k}\right)}$ matrix is the attention layer of DGAT. After network training, ${\boldsymbol{att}}_{\boldsymbol{ij}}^{\left(\boldsymbol{k}\right)}$ matrix records the weights between nodes in ST slice. We arrange the nodes in accordance with their spatial positions and color the edges based on the node weight relation matrix ${\boldsymbol{att}}_{\boldsymbol{ij}}^{\left(\boldsymbol{k}\right)}$, thereby realizing the detection of microenvironmental heterogeneity [24].

### Evaluation for clustering by benchmark comparison with baseline methods

We benchmarked STMGraph against 10 state-of-the-art tools (Leiden [17, 18], Louvain [18], STEEL [43], SpaCGN [20], GraphST [30], SpaceFlow [32], BayesSpace [19], DeepST [21], STAGATE [24], and Spatial-MGCN [28]), ensuring that the number of categories in each slice partition matched the number of manually labeled categories. The latent embeddings generated by nine tools are all clustered using the mclust [44] excluding Leiden and Louvain, and the clustering results were evaluated using adjusted Rand index (ARI) [45], normalized mutual info (NMI) [46], and Fowlkes–Mallows score (FMS) [47]. We ran 10 baseline methods in default parameter setting (see details in Supplementary Methods).

## Results

### DGAT demonstrated superior ability over static GAT in detecting the spatial heterogeneous similarities within tissue microenvironment

To compare the abilities of dynamic graph attention variant (DGAT) and static graph attention mechanism (GAT) on detecting of heterogeneous similarity among adjacent spots within tissue microenvironment, we conducted the ablation experiments using the datasets of coronal mouse brain section and mouse posterior brain section of 10× Visium datasets. We found that STMGraph via DGAT provides a more accurate description of the boundaries of coronal mouse brain sections such as the cortex, hippocampus, and midbrain cortex, especially the layer 1 region compared to STAGATE and STMGraph-w/o-D (Fig. 2A–C and E). In mouse posterior brain sections, STMGraph was better able to discern heterogeneous spatial similarity between coronal structures than STMGraph-w/o-D and STAGATE (Fig. 2F–H and J). Due to the presence of noise in the original spatial expression profile, STMGraph-w/o-M (without dual-mask) utilizing DGAT fails to rectify representations of nodes from two views, leading to irregular node correlation degrees (Fig. 2D and I). In the STMGraph-w/o-D model, the GAT struggled to capture the heterogeneity among different spots due to its limitation in dynamically querying the node representations that have undergone the dual-remasking process. The utilization of static attention in STAGATE hindered its ability to differentiate between contributions from neighboring points, thereby preventing the establishment of relationships between microscopic spots in the two mouse brain sections ultimately (Fig. 2E and J). The results indicated that DGAT’s spatial-context-awareness is more accurate than GAT in such intricate scenario, enabling STMGraph to consider heterogeneous similarity between adjacent spots without pre-clustering.

### Best performance of STMGraph than state-of-the-art tools from multi- to subcellular resolutions

(1) Spatial clustering performance of STMGraph on 10× Genomics Visium datasets

We evaluated the spatial clustering performance of STMGraph on the LIBD human dorsolateral prefrontal cortex (DLPFC) dataset (Supplementary Table S1) generated using 10× Genomics Visium [48]. The dataset contains spatially resolved transcriptomic maps of 12 DLPFC slices, with each depicting the human dorsolateral prefrontal cortex and white matter [48]. We benchmarked STMGraph against 10 state-of-the-art tools including Leiden [18], Louvain [18], STEEL [43], SpaCGN [20], GraphST [30], SpaceFlow [32], BayesSpace [19], DeepST [21], STAGATE [24], and Spatial-MGCN [28] (supplementary Table S3). Among the comparisons, STMGraph achieved the highest median ARI of 0.577, FMS of 0.664, and NMI of 0.689 for 12 slices (Fig. 3A, Supplementary Materials, Fig. S1 and Table S4) and the lowest variance score among all tools (excluding conventional clustering) followed by Spatial-MGCN in performance. Besides, STMGraph also achieved the best clustering results when benchmarking of simulated data [49] (Supplementary Materials and Fig. S2). Among the evaluated algorithms, there was significant variance in ARI scores for the 12 DLPFC slice dataset, with exceptions noted for the images generated by GraphST and Spatial-MGCN. The distinction lines among clusters generated by the other algorithms were not sufficiently smooth. The reason for the smooth distinction lines of GraphST images was the rigorous selection of positive and negative examples; however, it may suffer from over-smoothing issues. The data preprocessing strategy of Spatial-MGCN involved removing unmarked spots before model training [28, 29]. The unclear positional information for unmarked spots made it difficult to assign them to specific categories manually. Although deleting these data can improve accuracy, in practical applications, where data were typically unmarked, we cannot filter spots based on known classification information. STMGraph achieved the highest ARI evaluation score of 0.69 for the DLPFC 151674 slice dataset (Fig. 3B and C). The clustering results obtained from each tool were further utilized for UMAP and PAGA analysis (Fig. 3D). After the dimensionality reduction of UMAP, spots of same category exhibited a higher degree of clustering by STMGraph.

To illustrate the enhanced performance of ST clustering achieved through the integration of the DGAT and MASK-REMASK algorithms in STMGraph, we conducted the ablation experiments utilizing the DLPFC dataset. Leveraging the strengths of DGAT in nodes, link, and graph prediction, STMGraph employing DGAT framework effectively enhanced the quality of latent embeddings, making it more significant boosting clustering ultimately. Moreover, the MASK-REMASK algorithm in STMGraph also facilitated enhancements in clustering performance (Supplementary Materials and Fig. S3A).

To evaluate the generalization abilities of STMGraph on 10× platforms, we benchmarked STMGraph against four tools on the datasets of mouse brain anterior [30] and human breast cancer [50] (Supplementary Table S1). The maximum ARI scores for the datasets of mouse brain anterior and human breast cancer were calculated as 0.435 and 0.618 (Fig. 3H), respectively, with both FMS and NMI securing the highest values when compared to all other tools evaluated (see Supplementary Materials and Fig. S4A and B). In addition, by utilizing scaled cosine error as the loss function, we revealed that a larger scaling factor is more suitable for slices with complex tissue types (Supplementary Fig. S5). We performed SDEG analysis [18] on the human breast cancer dataset based on STMGraph clustering, which correctly distinguished IDC_4, IDC_1, IDC_5, DCIS/LCIS_5, IDC_2, Healthy_1, and Tumor_edge_1 (Fig. 3H). The top two to three differentially expressed genes for each of six regions were also shown (Fig. 3E). Several genes particularly *CXCL14*, *COX6C*, *CRISP3*, and *MPG* with high expression levels in these tumor regions were identified, while healthy cells exhibited few significant genes, with only *MALAT1* observed (Fig. 3E). The *CXCL14*, *COX6C*, and *CRISP3* have been verified are highly expressed genes of invasive dual carcinoma (IDC) in breast cancer [51–53]. *MPG* has been identified as a highly expressed gene in human breast cancer tumors [54]. *MALAT1* is generally overexpressed in patient tumors and metastases [55]. High expression of *MALAT1* was detected in healthy tissues, which indicates a potential risk of tumor occurrence and infiltration (Fig. 3E). Simultaneously, cell–cell communication analysis using CellPhoneDB [56] indicated that healthy_1 exert minimal self-influence and exhibit strong interaction with neighboring cancer tissues (Tumor_edge3 and Tumor_edge1). Thus, the infiltration of healthy tissue was likely to be due to the influence of recent tumor cells. The weight of the cell communication diagram represents the total receptor–ligand pairs with healthy cells as ligands and the remaining cells as receptors. Cancer cells can modify their microenvironment through these signals, thereby promoting tumor progression (Fig. 3F). The findings implied that the suppression of *MALAT1* could potentially impede the infiltration and invasiveness of breast cancer cells. Besides, *IGFBP7* can impact the behavior of tumor cells through various mechanisms, including the induction of cell senescence and apoptosis, as well as the inhibition of tumor proliferation [57]. STMGraph categorized healthy regions into 8, 11, and 19, with *IGFBP7* showing high expression in healthy region 8 located far from cancer cells (Fig. 3E and G). It indicated that STMGraph is capable of distinguishing heterogeneous states in tissue based on the varying degrees of invasion by cancer cells, with region 8 exhibiting low levels of invasion, while other software such as GraphST and STAGATE are less accurate in their precise delineation of healthy cells (Supplementary Fig. S4). Overall, STMGraph exhibited remarkable proficiency in spatial domain recognition for different datasets on 10× platforms, therefore providing the potential prediction ability to finding biological meaningful candidate genes for downstream validation and applications in oncology research.

(2) Spatial clustering evaluation of STMGraph on Slide-seqV2, Stereo-seq, and STARmap datasets

**Figure 4:**
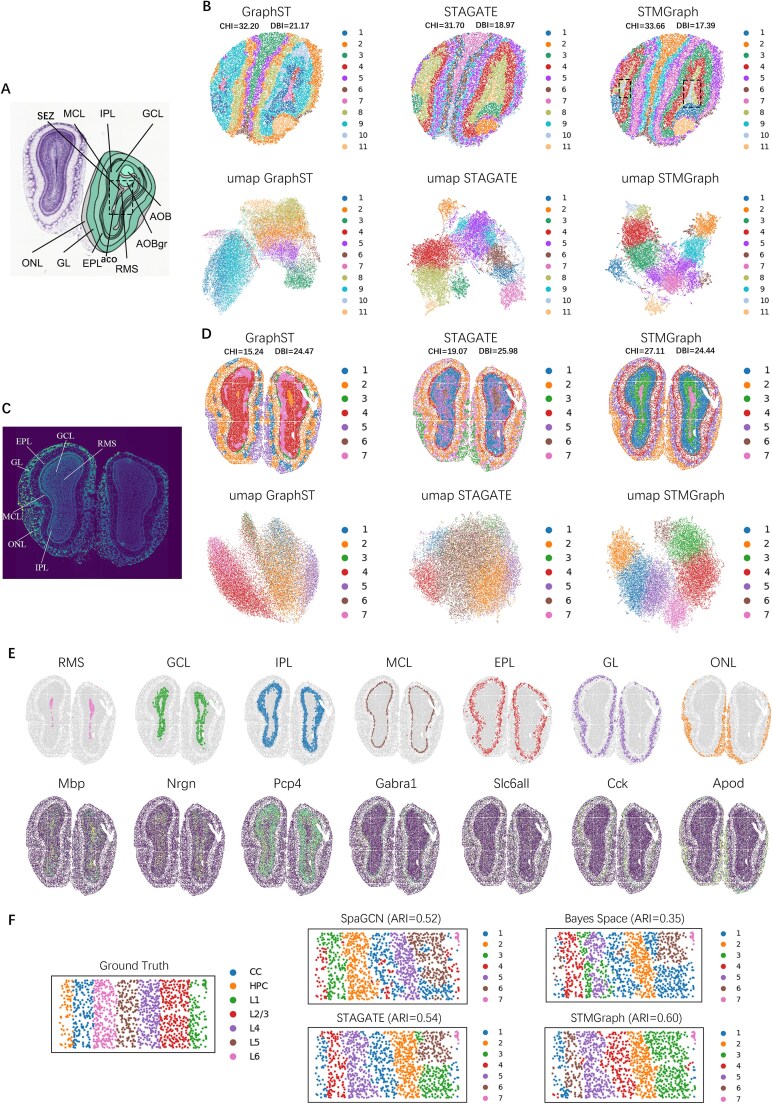
Performance of STMGraph for dataset of mouse olfactory bulb (MOB) from Stereo-seq, Slide-seqV2, and STARmap platforms. (A) Laminar organization of MOB annotated by the Allen reference atlas. (B) Spatial domains generated by GraphST, STAGATE, and STMGraph embeddings in the MOB tissue section generated by Slide-seqV2, and their corresponding UMAP representations. (C) Laminar organization of MOB annotated in the DAPI-stained image generated by Stereo-seq. (D) Spatial domains generated by GraphST, STAGATE, and STMGraph embeddings in the MOB section generated by Stereo-seq, and their corresponding UMAP representations. (E) The clustering results of GraphST, STAGATE, and STMGraph on laminar organization of MOB. The visualization of the clusters detected by STMGraph and related layer-marker genes expression. (F) Manual annotations served as ground truth, and the spatial domains generated by SpaGCN, BayesSpace, STAGATE, and STMGraph embeddings for mouse visual cortex dataset generated by the STARmap.

Benchmarking test of STMGraph against other software were also performed based on datasets generated by three ST platforms, Stereo-seq [6, 7], Slide-seqV2 [5], and STARmap [8], with single-cell resolution. The STAGATE, GraphST, and STMGraph that support these platforms were compared. Firstly, STMGraph identified laminar organization in mouse olfactory bulb tissue sections, sequenced by Stereo-seq and Slide-seqV2. STMGraph was able to discriminate the rostral migratory stream (RMS) of mouse olfactory bulb tissue with finer resolution, accurately dividing RMS into subependymal zone and anterior commissure of olfactory limb (aco) as indicated by the black dashed box (Fig. 4A). STMGraph obtained the highest ARI score in the clustering of datasets generated from Stereo-seq of mouse embryos (Supplementary Fig. S6). Lastly, the clustering performance of STMGraph was also the best, achieving the highest ARI score (0.60) for the dataset of the mouse visual cortex [8] generated by STARmap (Fig. 4F; Supplementary Table S1).

STMGraph was further used to identify laminar structures containing the RMS, granule cell layer, internal plexiform layer, mitral cell layer, external plexiform layer, and olfactory nerve layer in slices of mouse olfactory bulbs (MOB) tissue generated by Stereo-seq and Slide-seqV2 [6, 58]. A larger Calinski–Harabasz index [59] and a smaller Davies–Bouldin index [60] indicated that STMGraph clustering results exhibit greater intraclass tightness and interclass separation. We observed that the RMS region was clustered in a more refined manner by STMGraph than by GraphST or STAGATE (Fig. 4B, within the black dashed lines), which closely resembles real tissue organization (Fig. 4A). Moreover, the analysis of MOB generated by Stereo-seq [6, 58] showed that STMGraph effectively separated these laminar regions and produced better results than others (Fig. 4D). Additionally, the clustering results of MOB through STMGraph matched well the expression patterns of maker genes (Fig. 4E). In the UMAP plot after clustering with STMGraph, points of different classes were distinctly separated and formed tighter and clearer clusters than in other tools (Fig. 4B and D). Furthermore, when analyzing the mouse visual cortex dataset generated by STARmap, we found that STMGraph exhibits excellent separation of different tissue regions and achieves the highest ARI score (Fig. 4F). The above satisfactory results indicated the effective ST-context-aware of STMGraph by successful separating and classifying the regions in tissues.

### STMGraph corrects batch-effects of embeddings to integrate multiple slices

**Figure 5:**
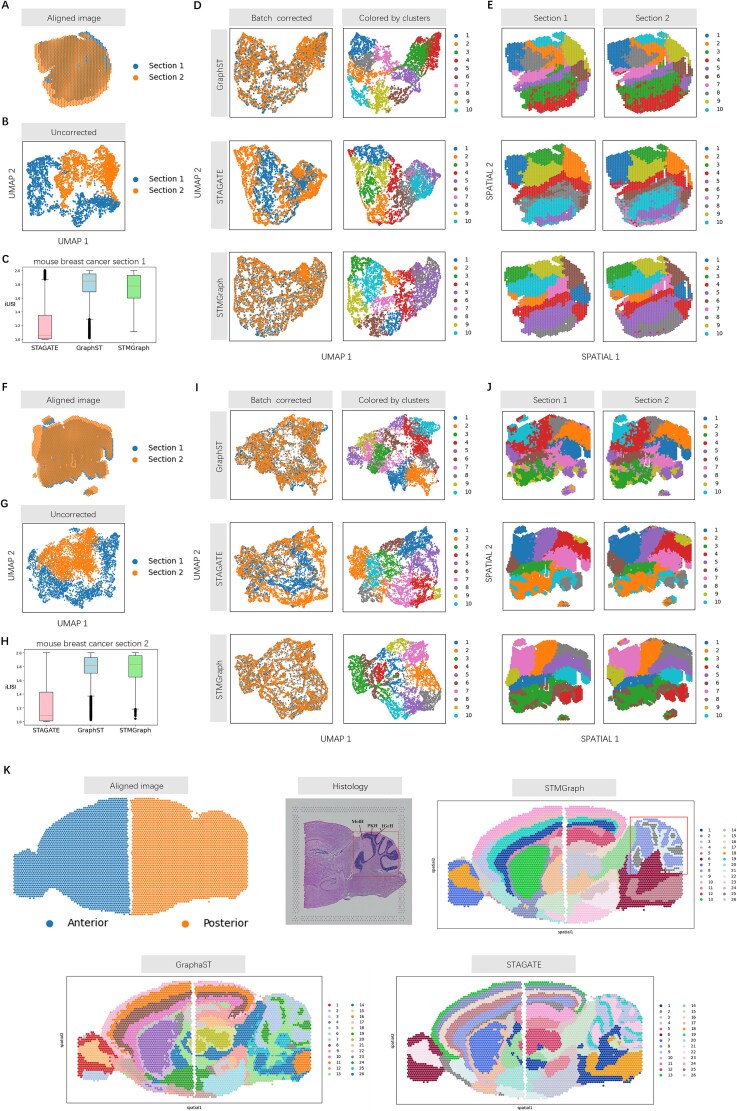
Batch-effects correction of embeddings for multi-slice integration. (A) The first set of mouse breast cancer images aligned with PASTE. (B) The first set of UMAP plots for mouse breast cancer samples was generated before batch-effects correction. (C) Box plot of iLISI metric for batch-correction results from different methods on the first set of samples. (D, E) UMAP plots after batch-effects correction for the first dataset and spatial clustering results from GraphST, STAGATE, and STMGraph. (F) The second set of mouse breast cancer sample images aligned by PASTE. (G) The second set of UMAP plots for mouse breast cancer samples was generated before batch-effects correction. (H) Boxplot of iLISI metric for batch-correction results from different methods on the second set of samples. (I, J) UMAP plots after batch-effects correction for the second dataset and spatial clustering results from GraphST, STAGATE, and STMGraph. (K) Horizontal integration results with mouse brain samples, which consists of anterior and posterior brain sections. PkH, MolH, and IGrH are annotations of mouse brain samples. The results include the classification of GraphST, STAGATE, and STMGraph.

**Figure 6:**
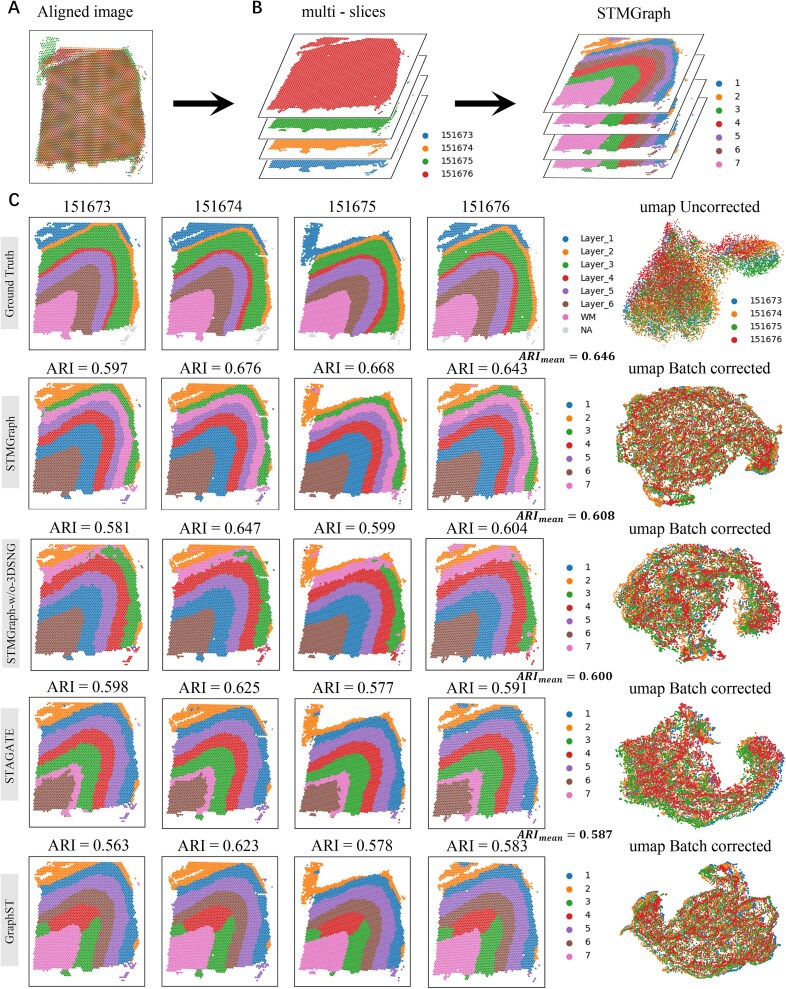
Spatial domain identification after multi-slice alignment of DLPFC dataset (151673–151676). (A) Aligning the centers of four sets of ST data and (B) presenting the 3D visualization outcomes. (C) Cluster analysis and UMAP visualization of STMGraph, STMGraph-w/o-3DSNG, STAGATE, and GraphST after integrating four sets of DLPFC data.

STMGraph incorporated the function of batch-effects correction for clustering of combined ST data of vertical slices. Differences in the UMAP plots of sections 1 and 2 of mouse breast cancer slices (Supplementary Table S1; Fig. 5A and F) indicated the batch-effects between the two slices [30, 39] (Fig. 5B and G). After dimensionality reduction mapping with UMAP, the spots from the two slices were significantly and evenly mixed by STMGraph compared to STAGATE and GraphST (Fig. 5D and I). To evaluate the results of the batch-effects integration of STMGraph compared with advanced tools, iLISI (integration local inverse Simpson’s index) [61] was used for evaluating the dataset of mouse breast cancer. The median iLISI for STMGraph and GraphST were ~2, while STAGATE was ~1 (Fig. 5C and H), indicating that the vertical batch-effects correction was more reliable for STMGraph and GraphST (Fig. 5C). However, GraphST had more outliers in the first section of mouse breast cancer slice, and the iLISI value in the second section of slice was slightly lower than STMGraph (Fig. 5C and H). In the subsequent clustering, STMGraph was more continuous at the category boundaries (Fig. 5E and J). In general, STMGraph improved the clustering performance of mouse breast cancer tissue by batch correction of multiple slices. In the horizontally merged slices of mouse brain [62], STMGraph achieved the most accurate clustering of spots (especially in the region marked by red box in Fig. 5K) by correctly distinguishing the Purkinje cell layer of CbHCx (PkH), molecular layer of CbHCx (MolH), and internal granular layer of CbHCx (IGrH) tissue regions. To verify the impact of implicit batch-effects correction on multi-slice clustering, we used PASTE [39] to align and 3D integrate four ST slices from the annotated DLPFC dataset (151673–151676) (Fig. 6A and B). After clustering using GraphST, STAGATE, and the STMGraph algorithm, it was found that STMGraph can achieve the highest average ARI score of 0.646. From umap, STMGraph and GraphST can mix points from different samples more evenly. In order to demonstrate the superiority of 3D-SNG over two slices of ST data, we conducted experiments using STMGraph-w/o-3D-SNG of SNG and observed a decrease in accuracy in spatial domain discrimination (Fig. 6C). STMGraph can indeed improve the discrimination of spatial domains of integrated multi-slices through batch correction. The ablation experiment showed that dual-remask algorithm compensates for the deficiency of DGAT that can only consider the local ST region, implements cross-batch regions to assist in integrating ST contextual information, and ultimately improves the signal-to-noise ratio for latent embedding, which is the key for the performance of batch correction in STMGraph implicitly (Supplementary Fig. S3B and C).

## Discussion and conclusion

Current ST algorithms are solely based on the original gene expression features, and the graph attention models are inadequate in distinguishing ST dropouts [12, 24, 25], which leads to poor noise discrimination when learning latent representations. Our study developed a high-spatial-perception model, STMGraph, which combines a DGAT with a dual-remask algorithm to effectively address above issues, achieves efficient heterogeneous similarity of spots detecting, spatial domain clustering, and implicit batch correction of multi-slices, and forms a practical tool for diverse ST studies.

The STMGraph mainly benefits from link prediction of using the DGAT, which bolsters the comprehension of the graph architecture and can more accurately capture the microenvironmental heterogeneous relationships of ST neighbors compared to tools (e.g. STAGATE [24]) using static GAT. Dual-remask masks the original ST features of the lowest and highest dimensions, thereby promoting the DGAT to learn more meaningful ST spots. During the self-supervision of STMGraph, DGAT infers the dual-remasked embeddings by aware of ST contextual information dynamically, which facilitates shared features between two views. Systematically benchmarking STMGraph against 10 state-of-the-art methods on datasets from various platforms showed the best accuracy and robustness for spatial domain identification. Besides, the performance of the tools (e.g. STMGraph, STAGATE, and Spatial-MGCN) ignoring the histological images is superior than tools (SpaGCN and DeepST) considering them, indicating that the histological information might not be the key to improving spatial recognition, and improper utilization hinders embeddings for clustering [35]. Due to spatial limitations of ST sequencing chips, it is necessary to integrate data from multiple sections for analysis [30]. STMGraph outperformed GraphST in clustering after batch correction, particularly in vertical slice integration, which is more crucial for the 3D construction of ST. Generative learning–based clustering algorithms (e.g. STAGATE) lack the ability to handle batch-effects in executing multi-slice integration, owing to its ability to aggregate only local neighborhoods and ignoring information outside the neighborhood [24]. Comparatively, STMGraph benefits from the cross-batch regional effects of dual-remask processes, where each spot not only considers its own SNG but also other SNG spots, which significantly improve the ability of batch-effects correction implicitly.

Except for the primary tasks of our algorithm, STMGraph also revealed the advantage of enhancing the spatial representation of candidate genes. For example, the expression patterns of marker genes (e.g. *RASGRF2*, *NTNG2*) in DLPFC (151674) slice, as well as that of MADs-box genes (genes belong to AP1-like, AGL6-like families) in Orchid flower-buds (slide_1) slice, were highly enhanced after denoising by STMGraph (Supplementary Materials and Fig. S7). In addition, the outstanding clustering abilities of STMGraph will substantially enhance the accuracy of identifying significant SDEGs (Supplementary and Fig. S8D and E). After accurately clustering several organs in mouse olfactory bulb dataset, STMGraph not only identified 1041 SDEGs which overlapped with top 1100 genes detected by SparkX [63], but also successfully detected significant specific SDEGs (*Mobp* and *Sox11* in the RMS organ) without overlapping SparkX. Conversely, the genes (e.g. *Gramd1b* and *Psma2*) uniquely detected by SparkX lack spatial expression capability.

Overall, STMGraph showed prospects in microenvironmental heterogeneity detecting, spatial domain clustering, batch-effects correction, and downstream SDEG detection, trajectory inference, and gene denoising, therefore providing a handy tool for diverse ST studies. The data types of histological images and ST profiles are distinct, which leads to errors in integrating embeddings between them. Future work will address issues of converting and unifying these two data types.

**Key Points**

- We developed a STMGraph, which combines dual-remask (MASK-REMASK) algorithm with dynamic graph attention model (DGAT) for ST data analysis. The dual-remask masks the embeddings to establish the dual-decoding-view to share features mutually, while DGAT modify and predict the unmasked and masked embedding, respectively, by self-supervision, dynamically integrating two views of context to reduce the interference of ST dropouts.
- STMGraph comprises three modules: (1) microenvironmental heterogeneity depicting, (2) spatially informed clustering, and (3) batch-effects correction. Systematic benchmarking reveals the superior performance of STMGraph, characterized by high accuracy and robustness in spatial-context-aware across ST datasets ranging from multi- to subcellular platforms.
- The proposed STMGraph algorithm has the far-reaching application for dissecting the developmental architecture of organisms. It is platform independent, as well as superior in downstream analysis of spatial differently expressed genes, trajectory inference, and gene denoising, making it a desirable novel tool for diverse spatial transcriptomics studies.

## Supplementary Material

Supplementary_Material_241231_bbae685(12)

## Data Availability

The data used in this paper are all publicly available. Detailed information can be found in Supplementary Table S1.

## Code availability

The code for the STMGraph algorithm is deposited at https://github.com/binbin-coder/STMGraph.
