# Correction to: STMGraph: spatial-context-aware of transcriptomes via a dual-remasked dynamic graph attention model

**DOI:** 10.1093/bib/bbaf133

**Published:** 2025-03-29

This is a correction to: Lixian Lin, Haoyu Wang, Yuxiao Chen, Yuanyuan Wang, Yujie Xu, Zhenglin Chen, Yuemin Yang, Kunpeng Liu, Xiaokai Ma, STMGraph: spatial-context-aware of transcriptomes via a dual-remasked dynamic graph attention model, *Briefings in Bioinformatics*, Volume 26, Issue 1, January 2025, bbae685, https://doi.org/10.1093/bib/bbae685

The following errors were identified by the authors:

1. In the MASK-REMASK method on page 4, $\boldsymbol{A}\in{\left\{0,1\right\}}^{\mathrm{N}\times \mathrm{N}}$ was mistakenly written as $\boldsymbol{A}\in{\left\{0,1\right\}}^{\mathrm{N},1}$. The dimension of the adjacency matrix A should be n × n to correctly represent the connection relationship between nodes. The wrong dimension n × 1 causes the matrix to fail to represent the structure of the graph correctly, so that calculations based on that matrix (the attention model shown in the figure) cannot be performed properly. The error may have been caused by a conflict of software editing versions.

2. In the Dynamic graph attention model (DGAT) method on page 4, $\left(\mathrm{ie}.,{\boldsymbol{h}}_i^{(0)}={\boldsymbol{x}}_{\boldsymbol{i}},\forall i\in \left\{1,2,3,\dots, N\right\}\right)\!,\ \mathrm{the}\ k\textrm{-th}\ \left(k\in \left\{1,2,\dots, L-1,L\right\}\right)$ was mistakenly written as $\left(\mathrm{ie}.,{\boldsymbol{h}}_i^{(0)}={\boldsymbol{x}}_{\boldsymbol{i}},\forall i\in \left\{1,2,3, nN\right\}\right),\mathrm{the}\ k\textrm{-th}\ \left(k\in \left\{1,2,e,L-1,L\right\}\right)$. The error may have been caused by a conflict of software editing versions.

3. In the Dynamic graph attention model (DGAT) method on page 4, $\left(\mathrm{i}.\mathrm{e}.{\hat{\boldsymbol{h}}}_i^{(L)}={\boldsymbol{h}}_i^{(L)}\right)\!,\ \mathrm{the}\ k\textrm{-th}\ \left(k\in \left\{2,\dots, L-1,L\right\}\right)$ was mistakenly written as $\left(\mathrm{i}.\mathrm{e}.{\hat{\boldsymbol{h}}}_i^{(L)}={\boldsymbol{h}}_i^{(L)}\right)\!,\ \mathrm{the}\ k\textrm{-th}\ \left(k\in \left\{2,\mathrm{ht}L-1,L\right\}\right)$. The error may have been caused by a conflict of word editing versions.

These errors have been corrected.

