# Supplementary material for: STMGraph: spatial-context-aware of transcriptomes via a dual-remasked dynamic graph attention model
Source: Brief Bioinform. 2025 Jan 6;26(1):bbae685. doi: 10.1093/bib/bbae685 (PMC11704419; doi:10.1093/bib/bbae685)
Supplement: Supplementary_Material_241231_bbae685(12) [file supplementary_material_241231_bbae685(12).pdf]

STMGraph: Spatial-context-aware of transcriptomes via a dual-remasked dynamic graph attention model

Supplementary Materials

<u>Supplementary Results</u>

Clustering performance of STMGraph in comparison with ten state-of-the-arts tools on the 10x Visium dataset

We conducted benchmark test on the dataset of LIBD human dorsal prefrontal cortex (DLPFC) [1], which contains 12 slices. We compared STMGraph with ten state-of-the-arts tools (Leiden [2], Louvain [2], STEEL [3], SpaCGN [4], GraphST [5], SpaceFlow [6], BayesSpace [7], DeepST [8], STAGATE [9], and Spatial-MGCN [10]) (**Supplementary Figure S1**). The spatial domain clustering were evaluated by the ARI (adjusted rand index) [11], NMI (Normalized Mutual Info) [12], and FMS (fowlkes mallows score) [13] for these 12 slice sets **(Supplementary Table S4)**. We found that clustering results using Leiden, Louvain, STEEL, SpaCGN, GraphST, SpaceFlow, BayesSpace, and DeepST methods showed patterns of lack of clear boundaries among categories. The clustered groups using GraphST, Spatial MGCN, and STMGraph algorithms did not show fragmented results. STMGraph performed the best clustering results for each of 12 slices, with smooth boundaries among clustered groups, followed by Spatial-MGCN. Moreover, algorithms based on generative Graph SSL (STAGATE, DEEPST, Spatial-MGCN) generally perform better in clustering than algorithms based on contrastive Graph SSL (GraphST, SpaceFlow). We referred to the method in spatialglue [14] for constructing the simulated data. we then benchmarked STMGraph with the other software for clustering. STMGraph performed the best with high values in ARI of 0.96, FMS of 0.97, and NMI of 0.95 **(Supplementary Figure S2)**. In addition, we compared the clustering performance of STMGraph with STAGATE, SpaceFlow, GraphST on the datasets of Mouse Brain Anterior and Human Breast Cancer, respectively. STMGraph also achieved the highest clustering score (ARI=0.44, FMS=0.46, NMI=0.74 and ARI=0.62, FMS=0.65, NMI=0.71) (**Supplementary Figure S4A, B**).

Ablation experiments for STMGraph

To demonstrate that how the Dynamic Graph Attention mechanism (DGAT) and MASK-REMASK algorithm in STMGraph have improved the performance of spatial transcriptome clustering, we conducted the ablation experiments using the dataset of LIBD human dorsal prefrontal cortex (DLPFC) [1]. The only difference between STMGraph and STMGraph-w/o-M is that STMGraph-w/o-M lacks the MASK-REMASK algorithm. The results showed that the ARI score of STMGraph clustering was the highest (**Supplementary Figure S3A**), followed by STMGraph-w/o-M. The only difference between STMGraph and STMGraph-w/o-D was that STMGraph-w/o-D uses a Static Graph Attention algorithm, while STMGraph uses a Dynamic Graph Attention algorithm. In the clustering evaluation of 12 slices in DLPFC, the ARI of STMGraph was significantly higher than that of STMGraph w/o-D, indicating that the Dynamic Graph Attention algorithm is more robust to neighbor noise and enhances the ability of spatial domain recognition. Moreover, the clustering effect of STMGraph-w/o-M is equivalent to that of Spatial-MGCN. Finally, it also proved that the MASK-REMASK algorithm can help improve the clustering performance of ST data following the comparison between STMGraph-w/o-M and STMGraph.

To demonstrate the ability of the MASK-REMASK algorithm in STMGraph in implicitly correct batch-effects for joint clustering of multi-slices, we conducted experiments on two sets of mouse breast cancer samples. The median of iLISI (integration local inverse Simpson's Index) in the first dataset (**Supplementary Figure S3B**) is as follows $\text{iLISI}_{\text{stagate}} = 1.06$, $\text{iLISI}_{\text{STMGraph-w/o-M}} = 1.21$, $\text{iLISI}_{\text{STMGraph-w/o-D}} = 1.52$, $\text{iLISI}_{\text{STMGraph}} = 1.78$. The median of iLISI values for the second set of data (**Supplementary Figure S3C**) is as follows $\text{iLISI}_{\text{stagate}} = 1.09$, $\text{iLISI}_{\text{STMGraph-w/o-M}} = 1.36$, $\text{iLISI}_{\text{STMGraph-w/o-D}} = 1.42$, $\text{iLISI}_{\text{STMGraph}} = 1.83$. The experiment showed that compared with STMGraph, the batch correction ability of STMGraph-w/o-M (STMGraph without MASK-REMASK) was significantly reduced, and the iLISI_{media} of the two mouse breast cancer samples was reduced by about 0.6 on average. STMGraph exhibited an average increase of approximately only 0.3 in iLISI_{media} score compared to STMGraph-w/o-D in both groups of mouse breast cancer samples. We found that after removing the MASK-REMASK algorithm, the ability of batch-effects correction of STMGraph-w/o-M significantly decreased. Moreover, we found that when the Dynamic Graph Attention mechanism was switched to the Static Graph Attention mechanism, the iLISI evaluation

of STMGraph-w/o-D only slightly decreased. Therefore, the MASK-REMASK algorithm not only has a positive effect on spatial domain recognition, but also is the key to the batch effect correction ability of STMGraph.

The effects of different scaling factors in loss function on clustering results

The effect of STMGraph on clustering of DLPFC dataset, Mouse Brain Anterior dataset [5], and Human Breast Cancer dataset [5, 15] using different scaling factors were tested. We found that the clustering performance in DLPFC was better when the scale factor was 1 (**Supplementary Figure S5A**), and the clustering performance of the mouse brain front dataset and human breast cancer data was better when the scale factor was 3 (**Supplementary Figure S5B**, **C**). ST data often exhibit varying levels of complexity, influenced by the number of genes and cells. A larger γ value leads to a faster decrease in loss, making it more suitable for reconstructing complex types of spatial transcriptome slice data. For instance, the diagram structure of 10× data (DLPFC) is relatively simple, thus a choice of 1 is appropriate. On the other hand, for single-cell level or rich tissue types of slice data with complex diagram structures, a choice of 3 is more suitable. Therefore, STMGraph needs to select the larger scaling factors in multi-classification tasks.

Spatial clustering evaluation of STMGraph on Stereo-seq datasets

We used datasets generated from Stereo-seq of mouse embryos at E9.5, E10.5, E11.5, and E14.5 [16] **(Supplementary Table S1)**, respectively. In the ST sequencing chip used for this dataset, each spot is surrounded by its 8 nearest neighbors. This is different from the 10× Visium data, where each spot is surrounded by 6 nearest neighbors. The number of categories selected for clustering in STMGraph were 12, 16, 16, and 16, respectively, representing the number of categories in the original annotations **(Supplementary Figure S6A-D)**. In this study, STMGraph achieved the highest ARI evaluation score. Furthermore, in the E11.5 mouse embryo data, the results of clustering using STMGraph correspond to specific marker genes in organs such as dermomyotome, heart, connective tissue, Head mesenchyme, gastrointestinal tract, and mesenchyme **(Supplementary Figure S6D, E)**.

STMGraph denoises gene expression for better characterizing spatial expression patterns for plant and animal slices

STMGraph integrated a function of imputing gene expression patterns from raw expression profiles. We utilized STMGraph to reduce noise in slice 151674 of the DLPFC dataset, aiming to enhance the visualization of gene spatial patterns. We compared the expression of six layer-marker genes [17] of the raw data to those denoised ones by STMGraph in the DLPFC section 151674 **(Supplementary Figure S7A)**. STMGraph had excellent performance in terms of denoising raw gene expression patterns. For example, *RASGRF2* exhibited specific expression in layers 2 and 3, *NTNG2* showed layer-specific expression in layer 6, and *CTGF* displayed layer-specific expression in WM **(Supplementary Figure S7B-D)**. After denoising, genes with raw high expression such as *RASGRF2* maintained their significant expression characteristics, and the differential expression patterns of genes became more pronounced, consistent with previous reports [17], while the spatial expression in the original data was more confused. Moran's I and Geary's C [2, 4, 9, 18] is an evaluation index utilized to assess the degree of correlation between spatial location and genes. The closer the value is to 1, the stronger the spatial correlation of genes becomes. Compared with raw data and the STAGATE, the STMGraph proposed in this paper attains the highest median score on DLPFC (151674).

Compared with animal tissue sections, plant tissue sections often exhibit more and lager gaps, and even the probe position of the chip corresponds to the gap in the image **(Supplementary Figure S7E)** due to the effects of the cell wall [19, 20], resulting to the higher level of expression noise. Therefore, the datasets from slices of orchid *Phalaenopsis* flower buds [21] **(Supplementary Table S1)** were used for testing the expression denoising abilities of STMGraph in plant tissue. After gene denoising with STMGraph, most of the MADs-box genes were expressed in the correct locations of slices of *Phalaenopsis* flower buds [22]. Overall, the relative expression values of genes decreased in spatial domains where they were incorrectly expressed, while they increased in the correct locations **(Supplementary Figure S7G)**. PAXXG080090 and PAXXG045840 belong to AP1-like family, are highly expressed in the floral primordia and meristem [22, 23]. The relative expression values of these genes were significantly enhanced spatially in clusters 21 and 23, representing floral meristem and primordia after denoising **(Supplementary Figure S7F, H, I)**. PAXXG301780 belongs to the AGL6-like

family and was expressed in the tepal, column, flower meristem and primordium [22, 23]. The spatial expression patterns of these genes were enhanced in clusters 17, 20, 21 and 23 belong to the tepal, column, floral meristem and primordia after denoising **(Supplementary Figure S7F, H, I)**. PAXXG323200 belongs to the SEP-like family and exhibits low expression levels in pollinium and high expression levels in column [22, 23]. The gene was expressed in cluster 24 at the pollinium and cluster 18 and 20 at the column. After gene denoising, the relative expression of gene in cluster 24 decreased, while their expression in cluster 18 and 20 remained the high level **(Supplementary Figure S7F, H, I)**. PAXXG182380 belongs to the AG-like family and shows high expression in the column or stamen [22, 23]. It was expressed in clusters 18, 19, 20, and 24, which include the column and pollinium. After gene denoising, the spatial correlation of the genes became stronger, and the expression regions became more prominent **(Supplementary Figure S7F, H, I)**. Compared with raw data and the STAGATE, the STMGraph proposed in this paper attains the highest median Moran's I and Geary's C score on orchid *Phalaenopsis* flower buds **(Supplementary Figure S7I)**. In summary, STMGraph demonstrated its effectiveness in smoothing noise and enhancing spatial gene expression patterns profoundly, thereby lowering the disorganization of the gene expression profiles in the raw expression data.

Spatial association of spatially differentially expressed genes (SDEGs) detection by STMGraph for mouse olfactory bulb dataset

For SDEGs detection, we used the clustering information obtained by STMGraph for mouse olfactory bulb datasets from Stereo-seq platform [16] (**Supplementary Figure S8**). SPARKX is a commonly used tool for detecting spatially highly variable genes (SVGs) [24]. To test the performance of spatial genes detected by STMGraph and obtained by SPARKX, we compared the spatial correlation of SDEGs detected by STMGraph and SVGs detected by SPARKX respectively. Totally 1041 SDEGs were obtained from STMGraph and compared with the top 1100 SVGs genes detected from SparkX. A total of 803 overlapped genes (yellow part) were detected between STMGraph and SparkX (**Supplementary Figure S8A**). The red part represents the unique SDEGs genes detected by STMGraph (n=238), while the green part represents the unique SVGs genes detected by SparkX (n=297). We calculated the Geary's C and Moran's I values [4] for 803 overlapping genes

mentioned above, 238 unique genes detected by STMGraph, and 297 unique genes detected by SparkX. Geary's C and Moran's I are used to observe the correlation between genes and spatial positions. STMGraph detected specific genes with high Geary's C and Moran's I values, followed by genes with overlapping specificity between STMGraph and SparkX (**Supplementary Figure S8 B, C**). *Mobp* and *Sox11* are unique genes detected by STMGraph, while *Gramd1b* and *Psma2* are unique genes detected by SparkX. We found that STMGraph detected higher spatial significance of specific genes and had higher Gear's C and Moran's I values, indicating a stronger spatial association of SDEGs detected from STMGraph.

Supplementary Methods

Spatial domain identification by clustering and refinement

We used the mclust clustering algorithm [25] to identify spatial domains using STMGraph embeddings. If the type of self-point is different from the type of points in its local SNG region, and all points in that region belong to another category, the type of self-point will be automatically converted to points in the same category as the local SNG region.

Benchmarking comparison of STMGraph with baseline methods and evaluation for clustering

Both Scanpy-leiden and Scanpy-louvain [2], a widely-used library for single-cell transcriptomics analysis, have been expanded to accommodate ST data. As the direct selection of the desired number of clusters is not permitted, we selected an appropriate resolution between 0 and 1 that would produce a clustering result consistent with manually annotated clusters **(Supplementary Table S3)**.

STEEL [3] is unsupervised and manifold learning-based algorithmic. Principal component analysis (PCA) was performed with a set of 20 components. We set highly variable genes hvg = 3000, number of neighbors for inferring radius k=20, minimal spatial Gini coefficient gini = 0.5, beads = 0.0005, perp = 35, and genes = 0.005 **(Supplementary Table S3)**.

BayesSpace [7] uses a Bayesian model with a Markov random field to model ST data for clustering, utilizing both spatial and gene expression information. We followed the analysis tutorial for BayesSpace in its GitHub repository and used

the following parameters: nrep = 50,000 and gamma = 3, platform = “Visium,” and mode = “normal” **(Supplementary Table S3)**.

SpaGCN [4] was applied with the default parameter settings for spatial clustering and integration of horizontal ST datasets. Specifically, the "histology" parameter was set to "True”, and p was set to 0.05. The learning rate and maximum training time were set to 0.05 and 200, respectively **(Supplementary Table S3)**.

In SpaceFlow [6] genes with expression in fewer than three cells and cells with expression of fewer than 100 genes were excluded. Subsequently, the normalized expression was subjected to a multiplication by a scale factor of 10,000 and log-transformed using a pseudo-count of 1. For feature inputs, 3000 highly variable genes were chosen. The learning rate, training epochs, and regularized weight factor were established at 0.001, 1000, and 0.1, respectively.

In STAGATE [9] the top 3000 highly variable genes were first selected and then log-transformed and normalized according to library size. The parameter “alpha” was set to 0. The learning rate and training epoch were left at the default 0.0001 and 500, respectively **(Supplementary Table S3)**.

In DeepST [8] the computer requires the highest memory resources, and the model requires pre training. We selected the image information parameters that needed to be organized, and determined the appropriate number of clusters based on the resolution. The default number of iterations for pre training was 100, the default number of iterations for main training was 500, the convolutional neural network (CNN) type used was ResNet50, the number of principal component fractions to be retained in PCA was 50, the default truncation radius was 150, and the number of neighbors for each node during graph construction is 12 **(Supplementary Table S3)**.

In GraphST [5], the top 3000 highly variable genes were first selected and then log-transformed and normalized according to the library size. The learning rate and training epoch were left at the default 0.001 and 500, respectively **(Supplementary Table S3)**.

Spatial-MGCN [10] designs a multi view GCN encoder. The parameters were set to dropout = 0, lr = 0.001, weight_decay = 5e-4, alpha = 1, and beta = 10 **(Supplementary Table S3)**.

Generation of simulated data

Simulation data was generated using SpatialGlue [14] following a zero expansion negative binomial (ZINB) distribution. We constructed a spatial gene expression matrix with 1,296 spots and 1,000 genes each, while all the spots were divided into five groups.

Correction of the batch-effects of embedding to integrate multiple slices

PASTE [26] is a software specifically designed for aligning and integrating spatial transcriptome data. The coordinates of each point in each ST data need to be converted into their own CSV file, and the alignment method is mode = "center".

Multi-slices analysis may encounter batch-effects of embeddings, which can lead to clustering bias between multi-slices data points. We first use PASTE [26] to center align the slices, and then integrate multiple slices using scanpy.concat (). STMGraph implicitly solves this problem, which we termed its cross-batch regional effect. The cross-batch regional effect refers to the iterative training process where each self-spot not only considers local SNG spots but also randomly replaced spots. During the spot replacement mechanism of MASK-REMASK, the calculation process replaces a small proportion of data points in a slice with data points from all other slices. We used 2D SNG for horizontal slicing, while in vertical slicing analysis, 3D SNG was employed. The target spot aggregates information within its own neighborhood spots through STMGraph, cleverly integrating the information of each spot to achieve batch removal and better clustering. Finally, we evaluated and compared the latent embeddings generated by STMGraph, STAGATE, and GraphST using the iLISI [27] evaluation.

Regeneration of gene expression profiles by data denoising

STMGraph captures contextual information from masked SNG in ST data based on dual-masked DGAT, thereby preserving the most critical information in low-dimensional embeddings. DGAT is composed of a decoder and two encoders. The decoder can map important information from the high-dimensional gene expression profiles to a low-dimensional embeddings. Through the decoder, the information in the low-dimensional embeddings is restored to the

original high-dimensional embeddings. This process smooths out the expression of many noisy genes. After decoding and encoding the original gene expression matrix, the last embedding was used for reconstructing the denoised gene expression profiles based on the original expression matrix.

Identification of spatially differentially expressed genes (SDEGs) and spatially variable genes (SVGs)

To identify spatially differentially expressed genes (SDEGs) within each spatial domain clustered by STMGraph, we applied the Wilcoxon test using the SCANPY package [2], with an FDR threshold of 1% (adjusted using the Benjamin-Hochberg method) and |logFoldChange|⩾2.

SparkX [24] is a software used for detecting spatially variable genes (SVGs). It uses gene expression profiles and spatial information as input and provides the false discovery rate (FDR) values for each gene **(Supplementary Table S3)**. The top 1100 spatially variable genes SVGs were screened by SparkX [24] with the FDR < 0.01 as the threshold.

We used Moran's I [4] and Geary's C [4] to measure the the spatial autocorrelation of genes (SDEGs, or SVGs) detected by STMGraph and SparkX.

Analysis of Cell-Cell communication

We used cellphonedb [28] with the parameters of “statistical_analysis” and “Threshold=0.1” for Cell-Cell communication analysis. The weight of the cell communication diagram represents the total ligand-receptor pairs with “healthy_1” cells as ligands and the remaining cells as receptors (**Figure 3F**), representing the frequency of intercellular communication.

Spatial trajectory inference

We employed the PAGA algorithm [29] and diffusion pseudotime implemented in the SCANPY [2] package to depict spatial trajectories. This method can observe the gene expression patterns underlying the developmental trajectory of cells within each tissue section. The PAGA diagram is visualized by executing the scanpy.tl.PAGA () and scanpy.pl. PAGA_compare () functions.

Reference

1. Maynard KR, Collado-Torres L, Weber LM et al. Transcriptome-scale spatial gene expression in the human dorsolateral prefrontal cortex, *Nat Neurosci* 2021;24:425-436.
2. Wolf FA, Angerer P, Theis FJ. SCANPY: large-scale single-cell gene expression data analysis, *Genome Biol* 2018;19:1-5.
3. Chen Y, Zhou S, Li M et al. STEEL enables high-resolution delineation of spatiotemporal transcriptomic data, *Brief Bioinform* 2023;24 (2):bbad068.
4. Hu J, Li X, Coleman K et al. SpaGCN: Integrating gene expression, spatial location and histology to identify spatial domains and spatially variable genes by graph convolutional network, *Nat Methods* 2021;18:1342-+.
5. Long Y, Ang KS, Li M et al. Spatially informed clustering, integration, and deconvolution of spatial transcriptomics with GraphST, *Nat Commun* 2023;14 (1):1155.
6. Ren H, Walker BL, Cang Z et al. Identifying multicellular spatiotemporal organization of cells with SpaceFlow, *Nat Commun* 2022;13 (1):4076.
7. Zhao E, Stone MR, Ren X et al. Spatial transcriptomics at subspot resolution with BayesSpace, *Nat Biotechnol* 2021;39:1375-+.
8. Xu C, Jin X, Wei S et al. DeepST: identifying spatial domains in spatial transcriptomics by deep learning, *Nucleic Acids Res* 2022;50 (22):e131–1.
9. Dong K, Zhang S. Deciphering spatial domains from spatially resolved transcriptomics with an adaptive graph attention auto-encoder, *Nat Commun* 2022;13 (1):1739.
10. Wang B, Luo J, Liu Y et al. Spatial-MGCN: a novel multi-view graph convolutional network for identifying spatial domains with attention mechanism, *Brief Bioinform* 2023;24(5):bbad262.
11. Hubert L, Arabie P. Comparing partitions, *J Classif* 1985;2:193-218.
12. Shen H, Cheng X, Cai K. et al. Detecting the Overlapping and Hierarchical Community Structure in Networks. *Community Structure of Complex Networks*. Berlin, Heidelberg: Springer Berlin Heidelberg, 2013, 19-44.
13. Fowlkes EB, Mallows CL. A Method for Comparing Two Hierarchical Clusterings, *J Am Stat Assoc* 1983;78:553-569.
14. Long Y, Ang KS, Sethi R et al. Deciphering spatial domains from spatial multi-omics with SpatialGlue, *Nat Methods* 2024;21:1658-1667.
15. Buache E, Etique N, Alpy F et al. Deficiency in trefoil factor 1 (*TFF1*) increases tumorigenicity of human breast cancer cells and mammary tumor development in TFF1-knockout mice, *Oncogene* 2011;30:3261-3273.
16. Chen A, Liao S, Cheng M et al. Spatiotemporal transcriptomic atlas of mouse organogenesis using DNA nanoball-patterned arrays, *Cell* 2022;185:1777-+.
17. Zeng H, Shen EH, Hohmann JG et al. Large-Scale Cellular-Resolution Gene Profiling in Human Neocortex Reveals Species-Specific Molecular Signatures, *Cell* 2012;149:483-496.
18. Lv T, Zhang Y, Li M et al. EAGS: efficient and adaptive Gaussian smoothing applied to high-resolved spatial transcriptomics, *GigaScience* 2024;13:giad097.
19. Lieben L. Spatial transcriptomics in plants, *Nat Rev Genet* 2017;18:394-394.
20. Gurazada SGR, Cox KL, Czymmek KJ et al. Space: the final frontier - achieving single-cell, spatially resolved transcriptomics in plants, *Emerg top life sci* 2021;5:179-188.
21. Teo ZWN, Zhou W, Shen L. Dissecting the function of MADS-Box transcription factors in orchid reproductive development, *Front Plant Sci* 2019;10:1474.
22. Liu C, Leng J, Li Y et al. A spatiotemporal atlas of organogenesis in the development of orchid flowers, *Nucleic Acids Res* 2022;50:9724-9737.
23. Su C-l, Chen W-C, Lee A-Y et al. A modified ABCDE model of flowering in orchids based on gene expression profiling studies of the moth orchid *Phalaenopsis aphrodite*, *Plos One* 2013;8:e80462.
24. Zhu J, Sun S, Zhou XJGb. SPARK-X: non-parametric modeling enables scalable and robust detection of spatial expression patterns for large spatial transcriptomic studies. *Genome Biol* 2021;22:1-25.

25. Fraley C, Raftery AE. MCLUST: Software for Model-Based Cluster, *Analysis* 1999;16:297-306.
26. Zeira R, Land M, Strzalkowski A et al. Alignment and integration of spatial transcriptomics data, *Nat Methods* 2022;19:567-+.
27. Longo SK, Guo MG, Ji AL et al. Integrating single-cell and spatial transcriptomics to elucidate intercellular tissue dynamics, *Nat Rev Genet* 2021;22:627-644.
28. Efremova M, Vento-Tormo M, Teichmann SA et al. CellPhoneDB: inferring cell–cell communication from combined expression of multi-subunit ligand–receptor complexes, *Nat Protoc* 2020;15:1484-1506.
29. Wolf FA, Hamey FK, Plass M et al. PAGA: graph abstraction reconciles clustering with trajectory inference through a topology preserving map of single cells, *Genome Biol* 2019;20:1-9.
30. Wang X, Allen WE, Wright MA et al. Three-dimensional intact-tissue sequencing of single-cell transcriptional states, *Science* 2018;361:eaat5691.
31. Stickels RR, Murray E, Kumar P et al. Highly sensitive spatial transcriptomics at near-cellular resolution with Slide-seqV2, *Nat Biotechnol* 2021;39:313-319.

Supplementary Tables

Supplementary Table S1. Datasets analyzed in this paper.

**Organism**	**Tissue**	**Data Source**	**Dataset Dimensions**	**Protocol**
Human	Dorsolateral prefrontal cortex	Maynard et al.[1] (http://spatial.libd.org/spatialLIBD)	151507：spots × genes = 4226 × 33538	10x Visium
Human	Dorsolateral prefrontal cortex	Maynard et al.[1] (http://spatial.libd.org/spatialLIBD)	151508：spots × genes = 4384 × 33538	10x Visium
Human	Dorsolateral prefrontal cortex	Maynard et al.[1] (http://spatial.libd.org/spatialLIBD)	151509：spots × genes = 4789 × 33538	10x Visium
Human	Dorsolateral prefrontal cortex	Maynard et al.[1] (http://spatial.libd.org/spatialLIBD)	151510：spots × genes = 4634 × 33538	10x Visium
Human	Dorsolateral prefrontal cortex	Maynard et al.[1] (http://spatial.libd.org/spatialLIBD)	151669：spots × genes = 3661 × 33538	10x Visium
Human	Dorsolateral prefrontal cortex	Maynard et al.[1] (http://spatial.libd.org/spatialLIBD)	151670：spots × genes = 3498 × 33538	10x Visium
Human	Dorsolateral prefrontal cortex	Maynard et al.[1] (http://spatial.libd.org/spatialLIBD)	151671：spots × genes = 4110 × 33538	10x Visium
Human	Dorsolateral prefrontal cortex	Maynard et al.[1] (http://spatial.libd.org/spatialLIBD)	151672：spots × genes = 4015 × 33538	10x Visium
Human	Dorsolateral prefrontal cortex	Maynard et al.[1] (http://spatial.libd.org/spatialLIBD)	151673：spots × genes = 3639 × 33538	10x Visium
Human	Dorsolateral prefrontal cortex	Maynard et al.[1] (http://spatial.libd.org/spatialLIBD)	151674：spots × genes = 3673 × 33538	10x Visium

Human	Dorsolateral prefrontal cortex	Maynard et al.[1] (http://spatial.libd.org/spatialLIBD)	151675：spots × genes = 3592 × 33538	10x Visium
Human	Dorsolateral prefrontal cortex	Maynard et al.[1] (http://spatial.libd.org/spatialLIBD)	151676：spots × genes = 3460 × 33538	10x Visium
Human	Human breast cancer	https://support.10xgenomics.com/spatial-gene-expression/datasets/1.1.0/V1_Breast_Cancer_Block_A_Section_1	spots × genes = 3798 × 36601	10x Visium
Mouse	Coronal Mouse brain section	https://www.10xgenomics.com/datasets/adult-mouse-brain-section-1-coronal-stains-dapi-anti-neu-n-1-standard-1-1-0	spots × genes = 2903 × 32285	10x Visium
Mouse	Mouse posterior brain	https://support.10xgenomics.com/spatial-gene-expression/datasets	spots × genes = 3353 × 31053	10x Visium
Mouse	Mouse olfactory bulb	Chen et al.[16] (https://github.com/JinmiaoChenLab/SEDR_analyses)	cells × genes = 27106 × 19527	Stereo-seq
Mouse	Mouse visual cortex	Wang, X., et al.[30] (https://www.starmapresources.com/data)	cells × genes = 1207 × 1020	STARmap
Mouse	Mouse olfactory bulb	Stickels, R.R. et al.[31] (https://portals.broadinstitute.org/single_cell/study/slide-seq-study)	cells × genes = 53208 × 23264	slide-seqV2
Mouse	Mouse embryo	Chen et al.[16] (https://db.cngb.org/stomics/mosta/)	4 slices (E9.5 (bins × spots = 5913 × 23015), E10.5 (bins × spots = 18670 × 23436), E11.5 (bins × spots = 27455 × 23175), E14.5 (bins × spots = 92928 × 18582))	Stereo-seq
Mouse	Horizontal integration results with mouse brain samples	Long et al.[5] (https://zenodo.org/record/6925603#.YuM5WXZBwuU)	Anterior (spots × genes = 2825 × 32285)<br>Posterior (spots × genes = 3289 × 3228)	10x Visium

Mouse	Batch integration of the two mouse breast cancer datasets	Long et al.[5] (https://zenodo.org/record/6925603#.YuM5WXZBwuU)	Section 1 (spots × genes = 1868 × 32285)<br>Section 2 (spots × genes = 1950 × 32285)	10x Visium
Orchid flower	Slide 1	Liu et al.[22] (https://academic.oup.com/nar/article/50/17/9724/6696353#supplementary-data)	spots × genes = 2379 × 28903	10x Visium

Supplementary Table S2. The hyperparameter settings of STMGraph used in the paper.

**SNG style**	**SNG Radius determination**	**Training rounds**	**γ**	**Datasets**
2D SNG	k = 6, model = "KNN"	1000	1	single ST slice or horizontally merged slices data obtained from 10× Visium[1, 22]
2D SNG	k = 8, model = "KNN"	1000	3	Stereo-seq acquired datasets of mouse embryos[16]
3D SNG	k = 13, model = "KNN"	1500	1	For the 10× Visium vertical section joint data [1, 5]
2D SNG	radius = 50, model="Radius"	1000	3	datasets with cellular and subcellular resolution from Stereo-seq and Slide-seq [16, 32]
2D SNG	radius = 400, model="Radius"	1000	3	for STARmap data [31]

Supplementary Table S3. Software and algorithms in this paper.

Software	Source	URL
Scanpy	Wolf et al.[2]	https://github.com/scverse/scanpy
STEEL	Chen et al.[3]	https://steel-st.sourceforge.io/#running-steel
BayesSpace	Zhao et al.[7]	https://github.com/edward130603/BayesSpace
SpaGCN	Hu et al.[4]	https://github.com/jianhuupenn/SpaGCN
SpaceFlow	Ren et al.[6]	https://github.com/hongleir/SpaceFlow
STAGATE	Dong and Zhang.[9]	https://github.com/QIFEIDKN/STAGATE
DeepST	Xu et al.[8]	https://github.com/JiangBioLab/DeepST
GraphST	Long et al.[5]	https://github.com/JinmiaoChenLab/GraphST
Spatial-MGCN	Wang et al.[10]	https://github.com/cs-wangbo/Spatial-MGCN
SparkX	Zhu et al.[24]	https://github.com/xzhoulab/SPARK
LISI	Longo et al.[27]	https://github.com/immunogenomics/LISI
PASTE	Zeira et al.[26]	https://github.com/raphaelgroup/paste/tree/main

Supplementary Table S4. The benchmark comparisons between STMGraph and 10 other popular methods based on the evaluation of metrics ARI (Adjusted Rand index), FMS (Fawlkes Mallows Score), and NMI (Normalized Mutual Info) across datasets

Methods	Metrics	151507	151508	151509	151510	151669	151670	151671	151672	151673	151674	151675	151676	Median
Louvain	ARI	0.195	0.143	0.143	0.113	0.124	0.194	0.185	0.176	0.220	0.266	0.198	0.223	0.189
	FMS	0.341	0.313	0.341	0.305	0.402	0.484	0.406	0.393	0.357	0.393	0.354	0.363	0.360
	NMI	0.295	0.214	0.262	0.242	0.145	0.189	0.210	0.164	0.333	0.316	0.277	0.311	0.252
Leiden	ARI	0.211	0.144	0.144	0.155	0.098	0.180	0.226	0.188	0.190	0.255	0.208	0.269	0.189
	FMS	0.373	0.319	0.340	0.347	0.475	0.455	0.441	0.401	0.341	0.382	0.353	0.400	0.378
	NMI	0.286	0.208	0.254	0.253	0.116	0.202	0.206	0.162	0.319	0.307	0.272	0.354	0.253
BayesSpace	ARI	0.428	0.500	0.314	0.470	0.449	**0.595**	0.564	0.603	0.588	0.584	0.408	0.435	0.485
	FMS	0.538	0.596	0.473	0.595	0.640	**0.771**	0.683	0.703	0.661	0.652	0.514	0.529	0.618
	NMI	0.631	0.613	0.466	0.615	0.559	0.569	0.664	0.694	0.697	0.685	0.586	0.598	0.614
SpaGCN	ARI	0.423	0.341	0.410	0.398	0.219	0.339	0.499	0.531	0.340	0.361	0.412	0.287	0.379
	FMS	0.528	0.457	0.546	0.528	0.451	0.564	0.634	0.646	0.452	0.467	0.506	0.400	0.517
	NMI	0.538	0.433	0.533	0.523	0.346	0.436	0.591	0.610	0.499	0.519	0.514	0.475	0.516
STEEL	ARI	0.223	0.124	0.151	0.094	0.100	0.349	0.230	0.111	0.229	0.264	0.366	0.282	0.226
	FMS	0.369	0.304	0.351	0.319	0.421	0.579	0.435	0.364	0.369	0.390	0.469	0.407	0.380
	NMI	0.302	0.248	0.280	0.190	0.195	0.343	0.245	0.157	0.372	0.378	0.510	0.408	0.291
SpaceFlow	ARI	0.492	0.351	0.279	0.269	0.303	0.278	0.276	0.401	0.391	0.352	0.252	0.330	0.316
	FMS	0.533	0.580	0.382	0.420	0.527	0.513	0.462	0.544	0.494	0.460	0.370	0.436	0.478
	NMI	0.535	0.575	0.412	0.452	0.484	0.425	0.445	0.502	0.537	0.447	0.406	0.447	0.450
DeepST	ARI	0.524	0.428	0.441	0.472	0.370	0.405	0.534	0.496	0.539	0.528	0.545	0.546	0.510
	FMS	0.606	0.541	0.606	0.564	0.612	0.531	0.607	0.524	0.643	0.561	0.653	0.652	0.606
	NMI	0.650	0.575	0.635	0.607	0.608	0.527	0.635	0.536	0.697	0.621	**0.721**	**0.707**	0.628
GraphST	ARI	0.486	0.512	0.540	0.515	**0.588**	0.441	**0.614**	0.618	**0.636**	0.427	**0.621**	0.429	0.527
	FMS	0.477	0.562	0.583	0.571	**0.689**	0.627	0.693	0.680	0.667	0.510	0.630	0.512	0.605
	NMI	0.541	0.589	0.598	0.598	0.568	0.500	0.662	0.670	0.700	0.573	0.639	0.538	0.594
Spatial-MGCN	ARI	**0.629**	0.462	0.526	**0.516**	0.390	0.357	0.606	**0.774**	0.599	0.596	0.543	0.576	0.559
	FMS	**0.694**	0.559	0.633	**0.624**	0.615	0.588	**0.715**	**0.837**	0.681	0.665	0.617	0.646	0.639
	NMI	**0.739**	0.602	0.673	**0.665**	0.505	0.526	**0.723**	**0.752**	0.660	0.691	0.667	0.671	0.669

STAGATE	ARI	0.528	0.430	0.472	0.470	0.449	0.449	0.576	0.575	0.614	0.496	0.606	**0.597**	0.512
	FMS	0.584	0.513	0.480	0.586	0.643	0.485	0.691	0.687	0.677	0.510	0.657	0.655	0.614
	NMI	0.633	0.600	0.554	0.583	0.606	0.465	0.665	0.693	0.721	0.550	0.689	0.686	0.620
STMGraph	ARI	0.530	**0.538**	**0.564**	0.511	0.501	0.472	0.606	0.600	0.621	**0.691**	0.598	0.590	**0.577**
	FMS	0.609	**0.627**	**0.663**	0.621	0.670	0.665	0.715	0.703	**0.686**	**0.742**	**0.663**	**0.657**	**0.664**
	NMI	0.665	**0.660**	**0.693**	0.649	**0.623**	**0.574**	0.700	0.686	**0.733**	**0.774**	0.708	0.698	**0.689**

The highest average ARI, FMS and NMI in each column are bolded.

Supplementary Figures

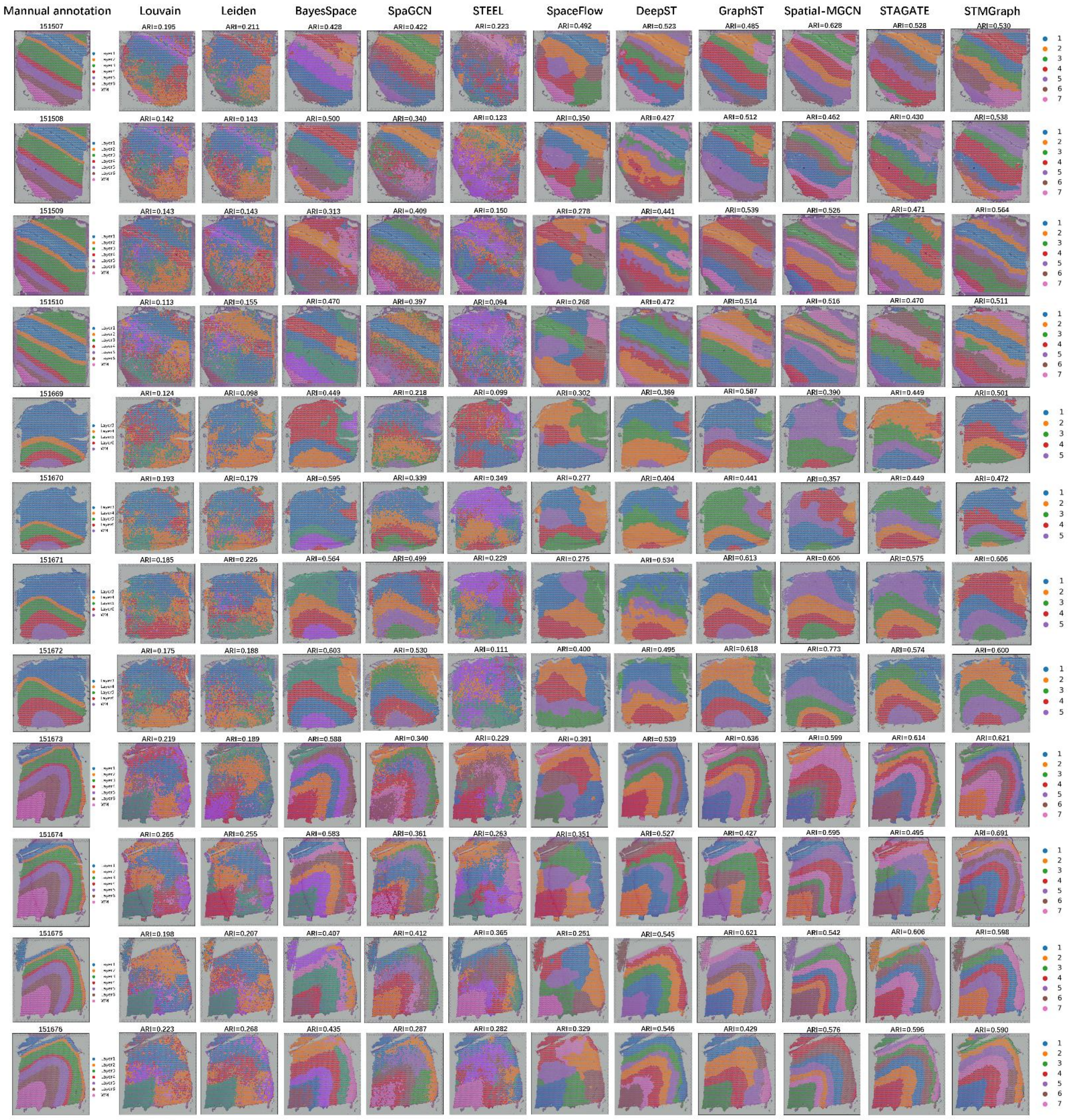

Supplementary Figure S1. Comparison of spatial domains by clustering assignments via Louvain, Leiden, BayesSpace, SpaGCN, STEEL, SpaceFlow, DeepST, GraphST, Spatial-MGCN, STAGATE, STMGraph, and manual annotation in all 12 sections of the DLPFC dataset.

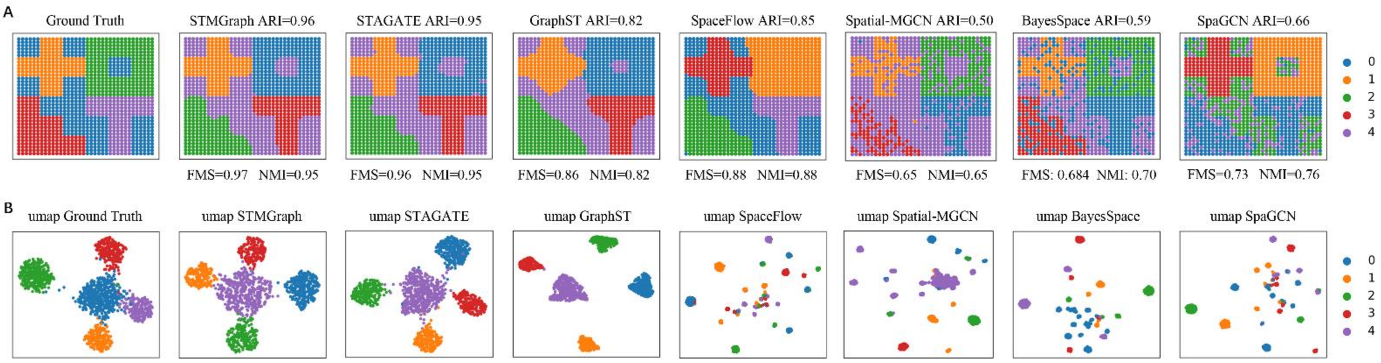

Supplementary Figure S2. Comparison of spatial domains by clustering assignments **(A)** and UMAP visualization **(B)** via ground truth, STMGraph, STAGATE, GraphST, SpaceFlow, Spatial-MGCN, BayesSpace, and SpaGCN in simulated data.

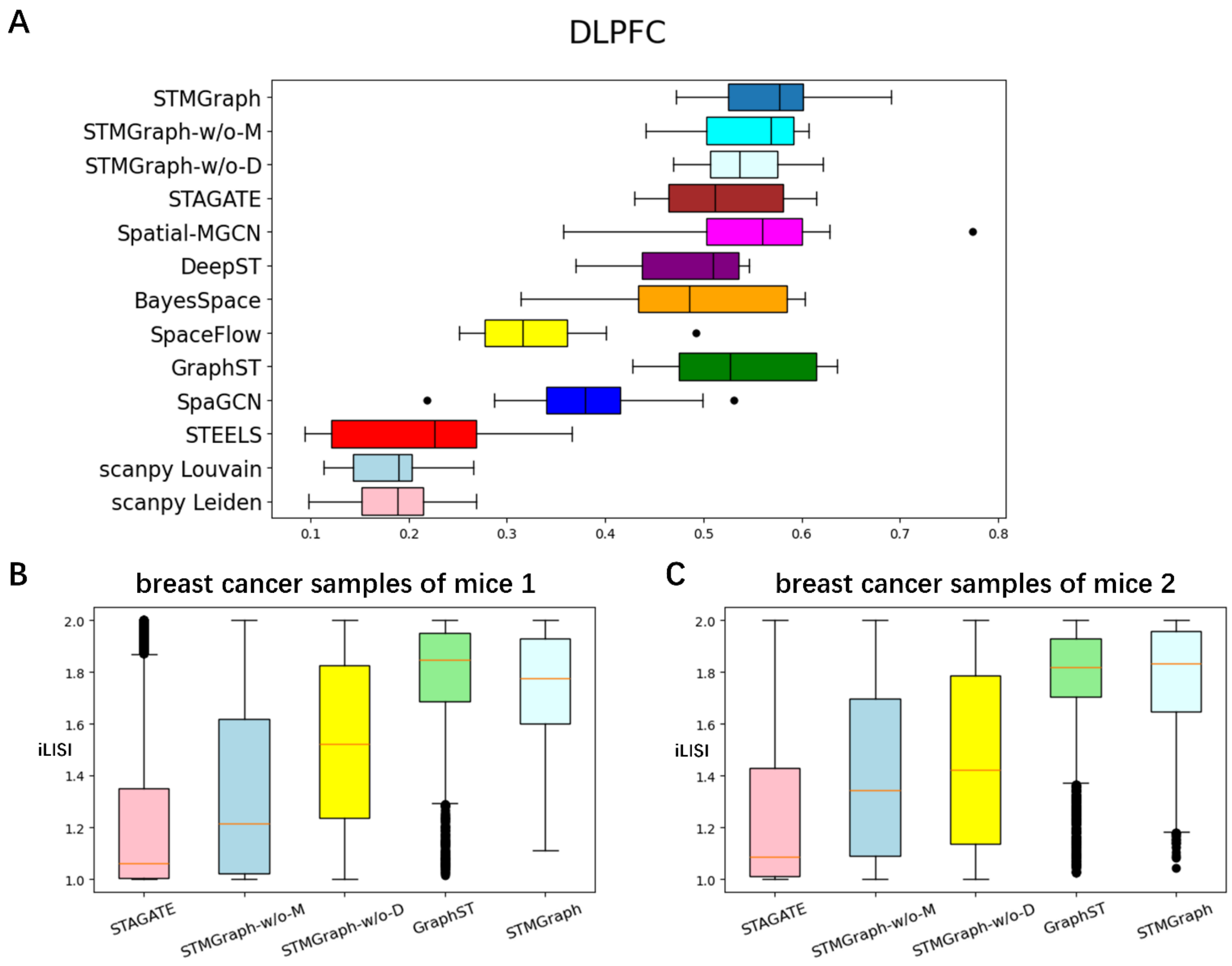

Supplementary Figure S3. Ablation experiment for STMGraph. The STMGraph-w/o-M algorithm is STMGraph that removes the MASK-REMASK algorithm and retains the DAGTE algorithm. The STMGraph-w/o-D tool is an adaptation of the STMGraph model where the dynamic graph attention mechanism is removed and replaced with static graph attention, while retaining the MASK-REMASK algorithm. **(A)** The adjusted rand index (ARI) scores of each tool on a DLPFC dataset with 12 slices. The iLISI (integration local inverse Simpson's Index) evaluation of breast cancer samples of mice in the first **(B)** and second **(C)** group.

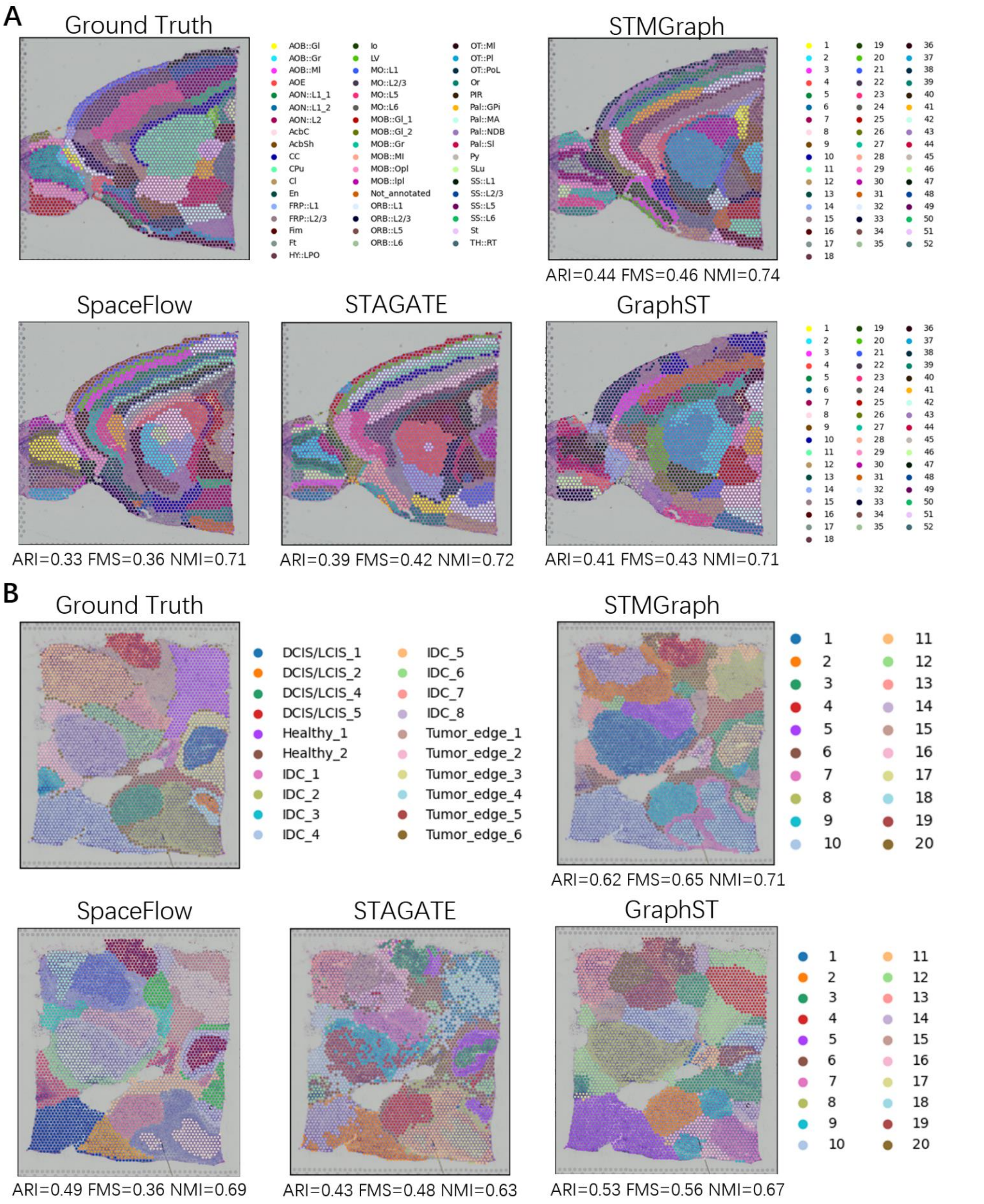

Supplementary Figure S4. Cluster analysis was performed on Mouse Brain Anterior dataset and Human Breast Cancer dataset. **(A)** Tissue domain annotations of Mouse Brain Anterior dataset. Clustering results of SpaceFlow, GraphST, STAGATE and STMGraph on the Mouse Brain Anterior dataset. **(B)** Tissue domain annotations of Human Breast Cancer dataset. Clustering results of SpaceFlow, GraphST, STAGATE and STMGraph on the Human Breast Cancer dataset.

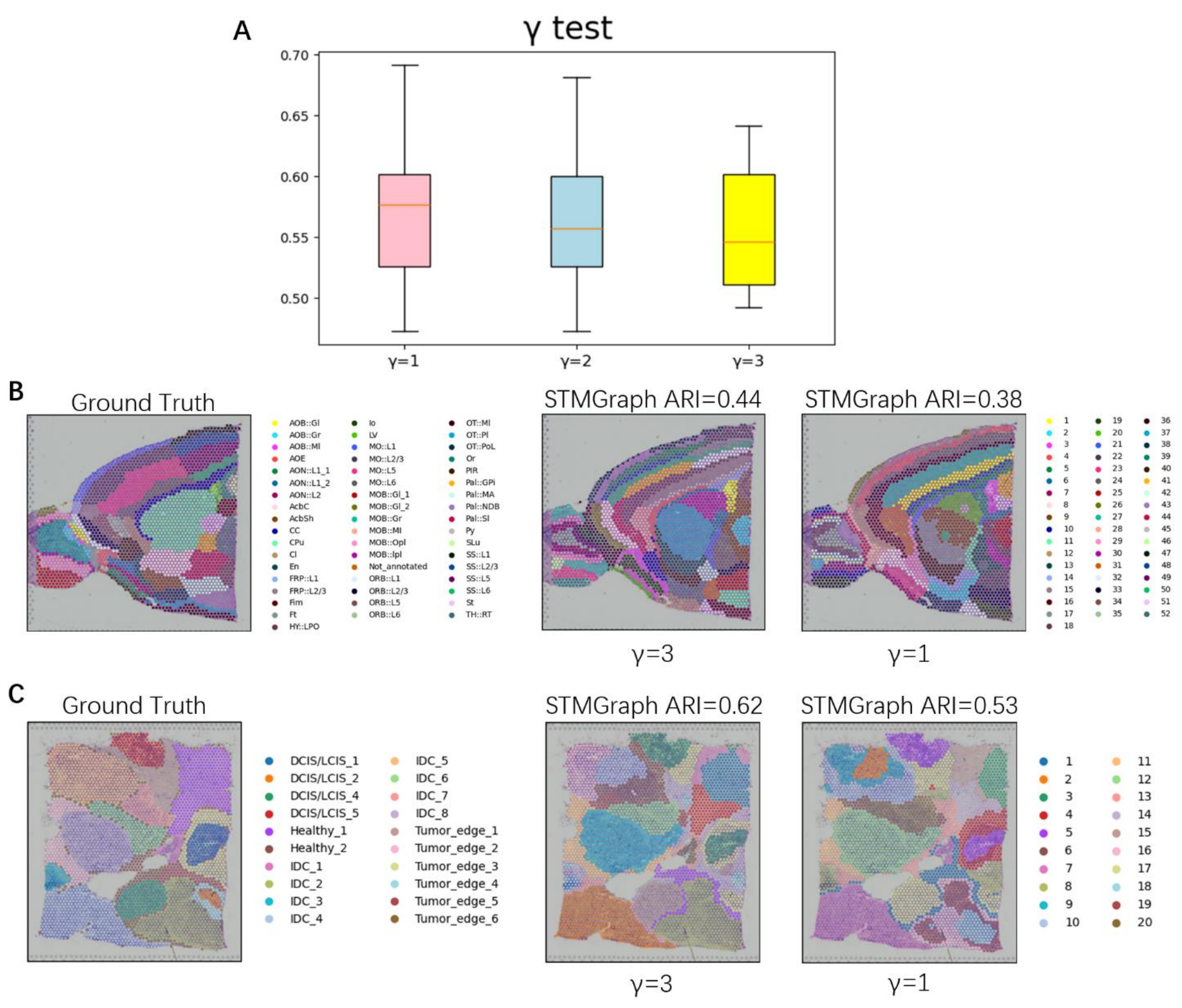

Supplementary Figure S5. Testing the effects of Scalable SCE Loss Functions with different γ Values (1 or 3) on clustering results using the DLPFC dataset (A), the Mouse Brain Anterior dataset (B), and the Human Breast Cancer dataset **(C)**.

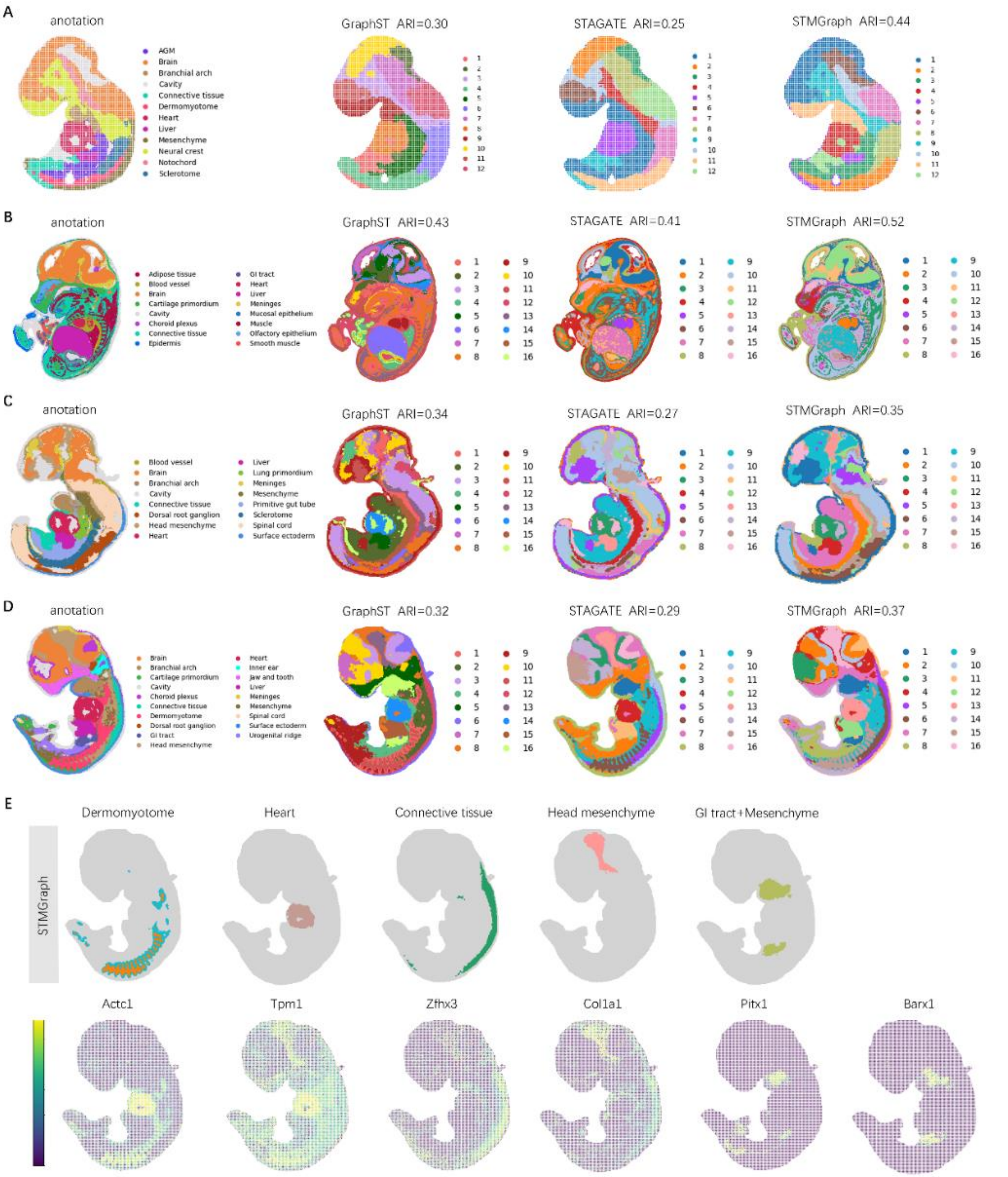

Supplementary Figure S6. Spatial clustering of ST data of mouse embryo using different methods. Tissue domain annotations and the clustering results of GraphST, STAGATE and STMGraph on the mouse embryo data of E9.5 **(A)**, E14.5 **(B)**, E10.5 **(C)**, as well as E11.5 **(D)** taken from the original Stereo-seq study. **(E)** Visualizations of the spatial domains identified by STMGraph along with the corresponding tissue-specific marker genes, respectivel

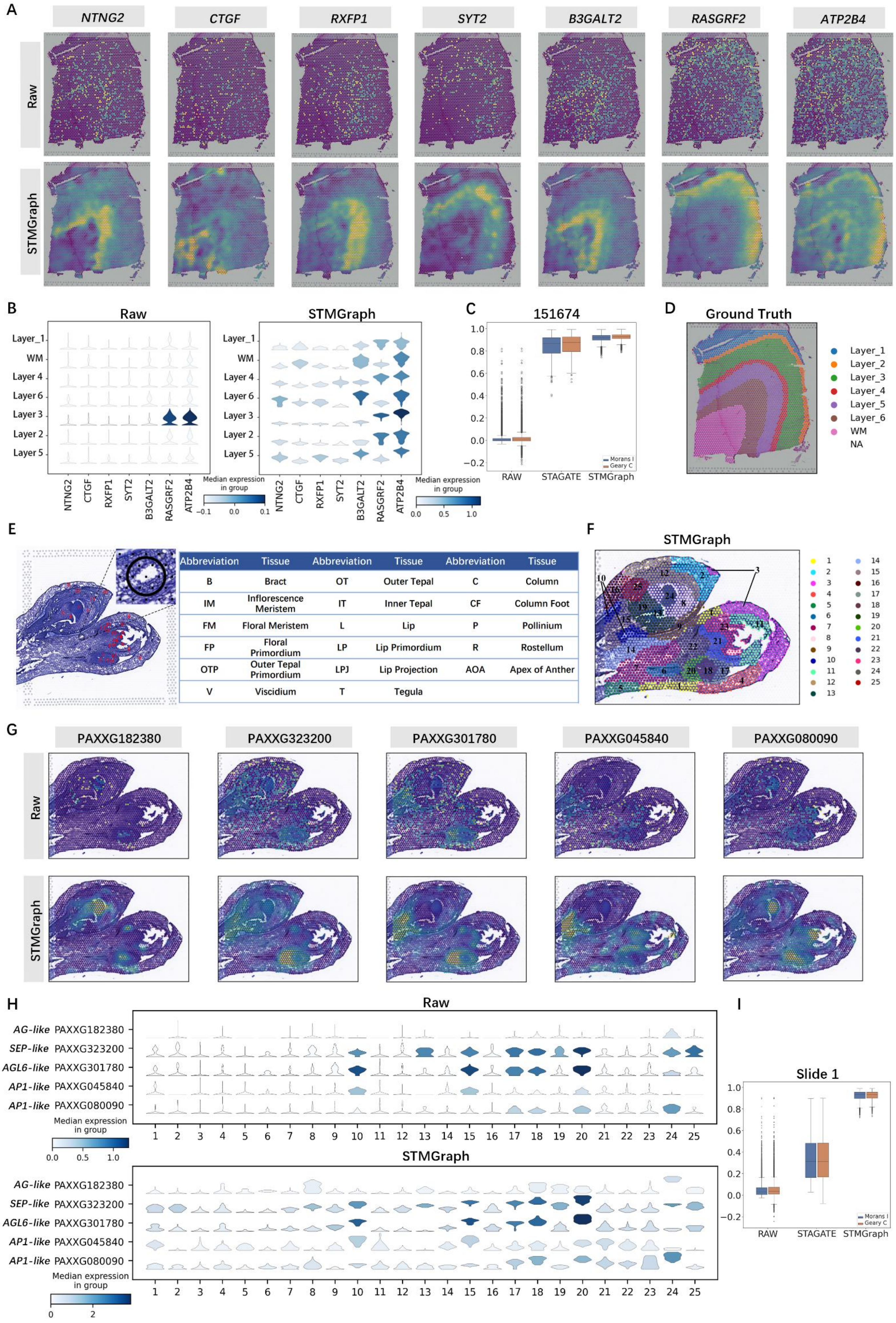

Abbreviation	Tissue	Abbreviation	Tissue	Abbreviation	Tissue
B	Bract	OT	Outer Tepal	C	Column
IM	Inflorescence Meristem	IT	Inner Tepal	CF	Column Foot
FM	Floral Meristem	L	Lip	P	Pollinium
FP	Floral Primordium	LP	Lip Primordium	R	Rostellum
OTP	Outer Tepal Primordium	LPJ	Lip Projection	AOA	Apex of Anther
V	Viscidium	T	Tegula		

Supplementary Figure S7. STMGraph imputes the expression patterns of marker genes in the human DLPFC and the MADs-box genes in slices of orchid *Phalaenopsis* flower buds. **(A)** The visualization of raw expression of layer marker genes and their expression patterns after STMGraph imputation. **(B)** The violin plot of raw expression and the expression imputed by STMGraph of cortical marker gene. **(C)** The Moran's I and Geary's C evaluation for the DLPFC raw data and DLPFC data that has been de-noised by STAGATE and STMGraph. **(D)** Manual annotation of the slice #151674 in DLPFC. **(E)** Annotation of Slide1 for orchid *Phalaenopsis* flower bud. **(F)** Cluster pattern diagram of STMGraph for Slide1 of orchid flower bud. **(G)** The visualization of raw expression of layer MADs-box genes and their expression patterns after STMGraph imputation. **(H)** The violin plot of raw expression and expression imputed by STMGraph for MADs-box genes in orchid *Phalaenopsis* flower buds. **(I)** The Moran's I and Geary's C evaluation for the raw data of orchid *Phalaenopsis* flower buds and data that has been de-noised by STAGATE and STMGraph.

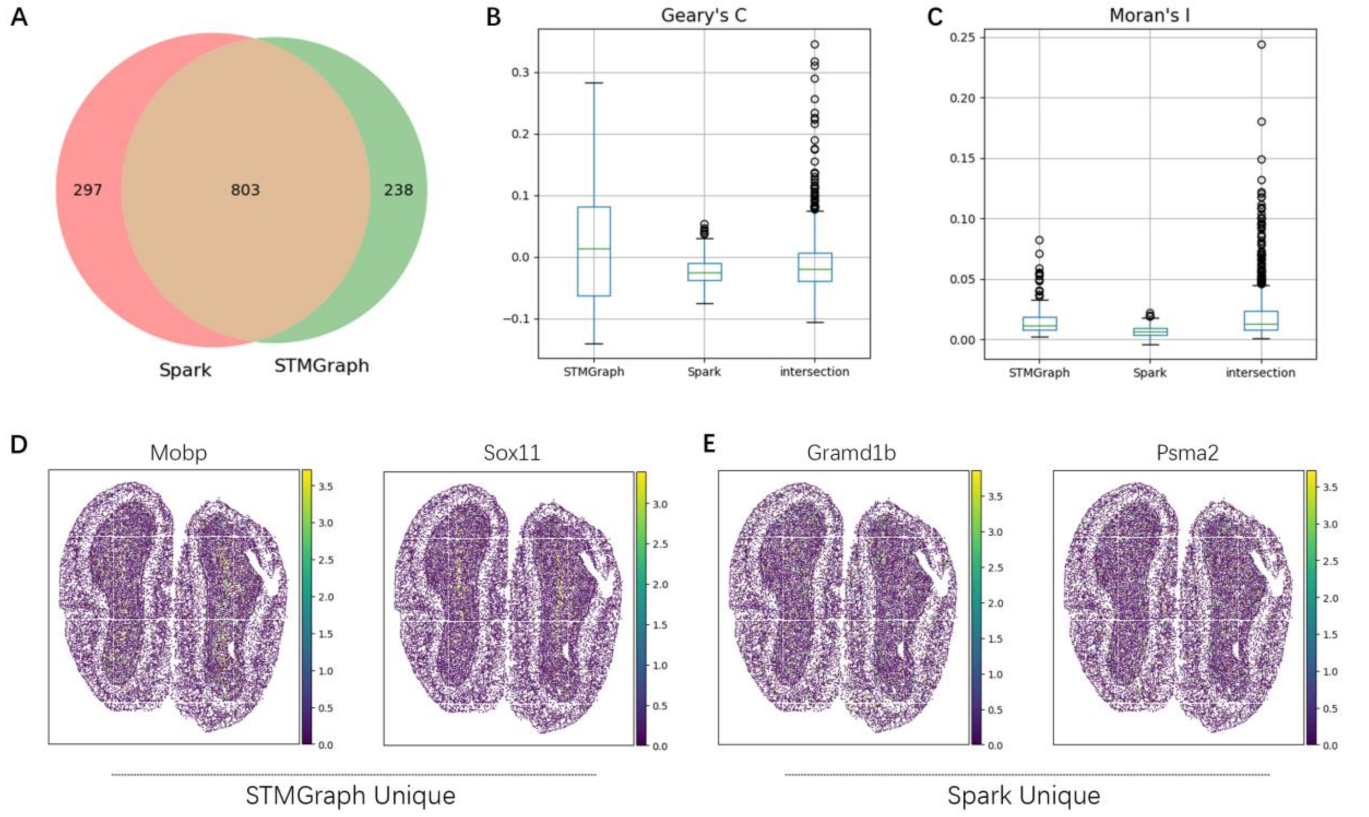

Supplementary Figure S8. Performance comparison of spatial differentially expressed genes (SDEGs) detection by STMGraph and spatially highly variable genes (SVGs) detected by SparkX for mouse olfactory bulb datasets. **(A)** SDEGs detected by STMGraph (n=1041) and SVGs detected by SparkX (top 1100), are showing in the Venn diagram. The red, green, and yellow areas are SparkX-specific genes, STMGraph-specific genes, as well as intersected genes detected by both STMGraph and SparkX. **(B)** The Geary's C evaluation for the STMGraph-specific genes, SparkX-specific genes, and intersected genes between them. **(C)** The Moran's I evaluation for the STMGraph-specific genes, SparkX-specific genes, and intersected genes between them. **(D)** Selected STMGraph-specific genes show spatial patterns significantly in RMS region of mouse olfactory bulb tissue. **(E)** Selected SparkX-specific genes show no/less spatial patterns in RMS region of mouse olfactory bulb tissue.
